# Intrinsically disordered region of Clr4/Suv39 regulates its enzymatic activity and ensures heterochromatin spreading

**DOI:** 10.1093/nar/gkaf878

**Published:** 2025-09-09

**Authors:** Rinko Nakamura, Aki Hayashi, Reiko Nakagawa, Yuriko Yoshimura, Naoki Horikoshi, Hitoshi Kurumizaka, Jun-ichi Nakayama

**Affiliations:** Division of Chromatin Regulation, National Institute for Basic Biology, Okazaki 444-8585, Japan; Basic Biology Program, Graduate Institute for Advanced Studies, SOKENDAI, Okazaki 444-8585, Japan; Division of Chromatin Regulation, National Institute for Basic Biology, Okazaki 444-8585, Japan; Laboratory for Cell-Free Protein Synthesis, RIKEN Center for Biosystems Dynamics Research, Kobe 650–0047, Japan; Division of Chromatin Regulation, National Institute for Basic Biology, Okazaki 444-8585, Japan; Laboratory of Chromatin Structure and Function, Institute for Quantitative Biosciences, The University of Tokyo, Tokyo 113-0032, Japan; Department of Cell Biology and Anatomy, Graduate School of Medicine, The University of Tokyo, Tokyo 113-0032, Japan; Laboratory of Chromatin Structure and Function, Institute for Quantitative Biosciences, The University of Tokyo, Tokyo 113-0032, Japan; Department of Biological Sciences, Graduate School of Science, The University of Tokyo, Tokyo 113-0032, Japan; Laboratory for Transcription Structural Biology, RIKEN Center for Biosystems Dynamics Research, Yokohama 230-0045, Japan; Division of Chromatin Regulation, National Institute for Basic Biology, Okazaki 444-8585, Japan; Basic Biology Program, Graduate Institute for Advanced Studies, SOKENDAI, Okazaki 444-8585, Japan

## Abstract

Methylation of histone H3 at lysine 9 (H3K9me), a hallmark of heterochromatin, is catalyzed by Clr4/Suv39. Clr4/Suv39 contains two conserved domains—an N-terminal chromodomain and a C-terminal catalytic domain—connected by an intrinsically disordered region (IDR). Several mechanisms have been proposed to regulate Clr4/Suv39 activity, but how it is regulated under physiological conditions remains largely unknown. We found that the N-terminus of Clr4 interacts with its C-terminal catalytic domain and represses its enzymatic activity. Detailed biochemical analyses revealed that basic amino acid residues in the IDR are involved in this interaction. Amino acid substitutions of these residues weakened this interaction, thereby promoting Clr4 activity *in vitro*. Interestingly, cells expressing mutant Clr4 with these substitutions showed a silencing defect, which suggested additional roles of the IDR *in vivo*. Genetic analysis revealed that the IDR functions in H3K9me spreading and that this activity is functionally linked to the RNAi pathway. We also showed that Clr4 binds to RNAs via the IDR and that RNA attenuates Clr4 autoinhibition *in vitro*. Furthermore, the IDR was found to contribute to the targeting of nucleosomal substrates *in vitro*. These results reveal a novel function of the Clr4/Suv39 IDR in regulating its enzymatic activity and heterochromatin spreading.

## Graphical Abstract

**Graphical Abstract:**
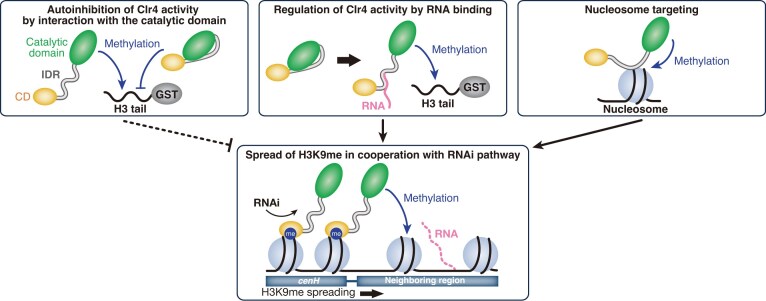

## Introduction

Heterochromatin, which comprises highly condensed chromatin structures, plays a pivotal role in maintaining genome stability and regulating gene expression in eukaryotes [1–3]. In many eukaryotes, including humans, histone H3 lysine 9 methylation (H3K9me)—catalyzed by the Clr4/Suv39 family of histone methyltransferases (HMTases)—is a hallmark of constitutive heterochromatin [4, 5]. The heterochromatin protein 1 (HP1) family recognizes H3K9me and facilitates chromatin condensation [6–8]. Once deposited, H3K9me is maintained during DNA replication, supported by several replication factors [9–13], and the heterochromatic state can be transmitted through mitosis and meiosis, resulting in epigenetic inheritance [14, 15].

In the fission yeast *Schizosaccharomyces pombe*, heterochromatin is formed at centromeres, telomeres, and the silent mating type region (*mat*) [16], and Clr4, a sole H3K9 methyltransferase in *S. pombe*, plays a critical role in heterochromatin assembly [6, 14]. Clr4 contains two conserved domains—an N-terminal chromodomain (CD) that can bind to H3K9me and a C-terminal catalytic domain consisting of the SET and associated domains—connected by an intrinsically disordered region (IDR) [4, 17]. This H3K9me binding of Clr4-CD is critical for H3K9me deposition on adjacent nucleosomes in a *cis*-acting manner, facilitating the spread of H3K9me at the heterochromatic region [18–20]. This mode of H3K9me spreading is referred to as the “read–write” mechanism. Increasing the affinity of Clr4 for H3K9me rescues the heterochromatin spreading defects caused by an H3 mutant [21]. The maintenance of ectopic heterochromatin introduced by Clr4 tethering is also dependent on this mechanism [22].

In addition to the read–write mechanism, the RNA interference (RNAi) pathway is critical for heterochromatin assembly [23], wherein the noncoding RNAs transcribed from heterochromatic repeats by RNA polymerase II are converted into small interfering RNAs (siRNAs) by the RNA-dependent RNA polymerase complex and the dicer ribonuclease (Dcr1) [24, 25]. The siRNAs are loaded onto the Argonaute protein (Ago1) of the RNA-induced transcriptional silencing (RITS) complex, and the RITS containing the siRNAs targets the heterochromatic repeats where the nascent RNAs are transcribed, presumably recruiting Clr4 along the way [26]. Deletion of the genes encoding the RNAi pathway components results in a defect in heterochromatin assembly at pericentromeric regions. While the RNAi pathway also targets the *mat* locus or telomeres, RNAi-independent mechanisms also contribute to heterochromatin assembly at these loci [27–29].

The enzymatic activity of Clr4 is tightly regulated in the cell, as promiscuous Clr4 activity induces aberrant gene silencing; several mechanisms have been proposed to regulate Clr4 activity [30]. Clr4 contains a short loop called the autoregulatory loop (ARL) in the C-terminal catalytic domain. The ARL inhibits the enzymatic activity of Clr4 by occluding H3 from the catalytic pocket in the SET-insertion domain, whereas this autoinhibition is alleviated by automethylation of lysine residues in the ARL [31]. Clr4 forms a complex, called the Clr4 methyltransferase complex (CLRC), and histone H3 lysine 14 ubiquitination (H3K14ub) catalyzed by the CLRC promotes Clr4 enzymatic activity [32, 33], with the Clr4 IDR connecting the CD and the catalytic domain contributing to H3K14ub recognition [32]. In the case of mammalian Suv39, an IDR near the N-terminus contributes to both catalytic activity and interaction with HP1 [20, 34]. In addition, the CD of Suv39 or its adjacent region has DNA/RNA-binding activity, which is important for its association with pericentromeric repeats [35–37]. However, how Clr4/Suv39 activity is regulated under physiological conditions is not fully understood.

Previously, we demonstrated a crosstalk between H3K14ub and H3K9me and also found that the Clr4 IDR, which connects the CD and the catalytic domain of Clr4, modulates Clr4 methyltransferase activity; N-terminally deleted mutant Clr4 showed a stronger activity against unmodified H3 than full-length Clr4 *in vitro* [32], suggesting Clr4 autoregulation involving its N-terminal region. In the present study, we focused on the IDR connecting the N-terminal CD and C-terminal catalytic domain of Clr4 and aimed to investigate its novel function in regulating Clr4 enzymatic activity and ensuring RNAi pathway-coupled heterochromatin spreading.

## Materials and methods

### Plasmid construction

To obtain Clr4 N-terminal recombinants, coding sequences for Clr4 (CD, 1–83, 1–104, 1–126, 1–191, 63–191, 63–126, 127–191) were PCR (polymerase chain reaction) amplified and cloned into the BamHI and EcoRI sites of pGEX6P-3. To obtain MBP (maltose-binding protein)-fused Clr4, coding sequences for Clr4 (full-length, Clr4-C) were PCR amplified and cloned into the BamHI and SalI (or HindIII for pMALc2-TEV) sites of pMALc2. To obtain Clr4 recombinants for HMTase assays, coding sequences for Clr4 (full-length, Clr4-C) were PCR amplified and cloned into the BamHI and HindIII sites of pCold-SUMO-TEV plasmid using NEBuilder HiFi DNA Assembly Mix (NEB, Ipswitch, MA, USA). To obtain plasmids to check Clr4 nuclear localization in yeast cells, coding sequences for Clr4 (WT, Mut1–3) were PCR amplified and cloned into the BamHI site of pREP41-EGFP (enhanced green fluorescent protein) plasmid using NEBuilder HiFi DNA Assembly Mix (NEB). To generate plasmids for *clr4* strain construction, 5× Flag-fused *clr4* coding sequence and the kanamycin resistance gene were cloned into pCRII-TOPO. All mutations in *clr4* were introduced using NEBuilder HiFi DNA Assembly Mix (NEB). Primers are listed in Supplementary Table S3. The plasmids for the production of a fusion protein comprising the N-terminal tail of *S. pombe* H3 (residues A1−K36) and glutathione S-transferase (GST) (H3N-GST), and GST-fused Clr4-C were prepared previously [32]. The plasmid for the *S. pombe* pericentromeric repeat was prepared previously [38].

### Generation of *S. pombe* strains

The *S. pombe* strains used in this study are described in Supplementary Table S4. To obtain *clr4* mutant strains, the DNA fragment containing the Flag-tagged *clr4* gene and the kanamycin resistance gene was amplified and inserted into the genome by homologous recombination. *clr4*Δ strains were generated by replacing the *clr4*^+^ gene with the kanamycin resistance gene by homologous recombination. The *ago1*Δ, *atf1*Δ, and *pcr1*Δ strains were generated by replacing their respective genes with the hygromycin resistance gene by homologous recombination.

### Antibodies

The following antibodies were used in this study: anti-FLAG M2 [F3165; 4 or 16 μg of antibodies were used for each ChIP (chromatin immunoprecipitation) or RNA-IP (RNA immunoprecipitation) experiment; Sigma–Aldrich, St. Louis, MO, USA], anti-H3K9me2 (mouse monoclonal antibody, 1:1300; kindly provided by Dr Takeshi Urano), HRP (horseradish peroxidase)-conjugated anti-FLAG M2 (A8592, 1:1000; Sigma), HRP-conjugated anti-Myc My3 (192-7, 1:1000; MBL, Tokyo, Japan), anti-tubulin (T5168, 1:1000; Sigma), and HRP-conjugated anti-mouse IgG (115-035-146; 1:20 000; Jackson ImmunoResearch, West Grove, PA, USA).

### Recombinant protein production and purification

Recombinant proteins for GST pull-down assays were prepared as follows: Recombinant GST-fused and MBP-fused proteins were expressed in BL21 (DE3) *Escherichia coli* and purified using Glutathione Sepharose 4B (Cytiva, Marlborough, CA, USA) and Amylose Resin (NEB), respectively, according to the manufacturer’s instructions. The eluted GST-fused proteins were further purified by anion-exchange chromatography [SOURCE 15Q, Cytiva; start buffer, 20 mM Tris–HCl (pH 7.6), 25 mM NaCl, 5% glycerol, 1 mM dithiothreitol (DTT); elution buffer, 20 mM Tris–HCl (pH 7.6), 1 M NaCl, 5% glycerol, 1 mM DTT]. The eluted MBP-fused proteins were dialyzed against dialysis buffer [1× phosphate-buffered saline (PBS), 10% glycerol, 1 mM DTT, 0.2 mM phenylmethylsulfonyl fluoride (PMSF)]. Clr4 used for cross-linking MS was prepared as follows: MBP-fused proteins were purified using amylose resin (NEB), according to the manufacturer’s instructions. The eluted proteins were cleaved with TEV protease overnight at 4°C. The cleaved proteins were further purified by anion-exchange chromatography [SOURCE15Q, Cytiva; start buffer, 20 mM Tris–HCl (pH 7.6), 25 mM NaCl, 5% glycerol, 1 mM DTT; elution buffer, 20 mM Tris–HCl (pH 7.6), 1 M NaCl, 5% glycerol, 1 mM DTT]. Clr4 recombinants used for HMTase assays and electrophoretic mobility shift assays (EMSAs) were prepared as follows: 6×His-tagged proteins were purified using TALON Metal Affinity Resin (Takara Bio, Kusatsu, Japan). The eluted proteins were cleaved by Ulp1 while being dialyzed at 4°C overnight. The cleaved tags and Ulp1 were removed by gel filtration chromatography in gel filtration buffer (50 mM Tris–HCl, pH 8.0, 150 mM NaCl, 1 mM DTT, 5% glycerol). All purified proteins were concentrated as needed in Amicon Ultra Spin Concentrators (Merck, Darmstadt, Germany), frozen in liquid nitrogen, and stored at −80°C until further use.

### Nucleosome reconstitution

Recombinant human histones H2A, H2B, H3, and H4 were overexpressed in *E. coli* and were purified as described previously [39]. A 167-bp Widom 601 DNA and purified histones were mixed and reconstituted by salt dialysis as described previously [39].

### *In vitro* GST pull-down assay

Glutathione Magnetic Agarose Beads (Thermo Fisher Scientific, Waltham, MA, USA) were incubated with 0.2 nmol of recombinant GST-fused Clr4 in 200 μl of binding buffer [125 mM Tris–HCl (pH 8.0), 150 mM NaCl, 0.05% Tween 20] at 4°C with rotation for >2 h. After washing, the beads were incubated with 0.2 nmol recombinant MBP-fused Clr4 and various amounts of pericentromeric RNA (379 nt single-stranded RNA) in 200 μl of reaction buffer [10 mM Tris–HCl (pH 7.5), 150 mM NaCl, 0.1 mM ethylenediaminetetraacetic acid (EDTA), 0.1% Triton X-100, 1× Complete EDTA-free (Roche Life Science, Basel, Switzerland)] at 4°C with rotation for 2 h. After washing, bound proteins were eluted by boiling in 20 μl of 1× SDS (sodium dodecyl sulfate) sample buffer and analyzed by SDS–polyacrylamide gel electrophoresis (SDS–PAGE) and coomassie brilliant blue (CBB) staining.

### Tritium-based *in vitro* HMTase assay

The substrate (6.2 μM of H3N-GST or 0.3 μM of nucleosomes) was mixed with 0.5 μM (for H3N-GST) or 2 μM (for nucleosomes) of Clr4 in 20 μl of reaction buffer [20 mM Tris–HCl (pH 8.0), 50 mM NaCl, 1 mM EDTA, 3 mM MgCl_2_, 0.1 mg/ml bovine serum albumin (BSA), 1 mM DTT, 1.1 μCi of S-(methyl-^3^H)-adenosyl-l-methionine (SAM) (Revvity, Waltham, MA, USA)] and incubated at 30°C for 1 h. Reactions were stopped by adding 4× SDS sample buffer, and the proteins were resolved by SDS–PAGE and visualized by autoradiography.

### Luminescence-based *in vitro* HMTase assay

The assays were performed with the various concentration of SAM, 100 nM of Clr4 and 50 μM of H3 peptide (ARTKQTARKSTGGKAPRKQL; GenScript, Piscataway, NJ, USA) in 20 μl of reaction buffer [20 mM Tris–HCl (pH 8.0), 50 mM NaCl, 1 mM EDTA, 3 mM MgCl_2_, 0.1 mg/ml BSA] at 30°C for 1 h. Reactions were stopped by adding trifluoroacetic acid to 0.1% and the production of S-adenosyl homocysteine was measured using the MTase-Glo Methyltransferase Assay Kit (Promega, Madison, WI, USA) and Nivo Microplate Reader (Revvity).

### *In vitro* transcription

*In vitro* transcription was performed as described previously [38], with some modifications. DNA fragments of *S. pombe* pericentromeric repeats or *ura4*^+^ gene were PCR amplified with a T7 promoter sequence and were used as templates for *in vitro* transcription with Thermo T7 RNA Polymerase (Toyobo, Osaka, Japan). Transcribed RNAs were purified by urea gel and stored at –25°C until further use.

### Electrophoretic mobility shift assays

EMSAs were performed as described previously [38], with some modifications. Different concentrations of recombinant Clr4 were incubated with 0.1 pmol of 379-nt single-stranded RNA or 379-bp double-stranded DNA corresponding to pericentromeric repeats or 0.25 pmol of reconstituted nucleosomes in 10 μl of buffer [20 mM Tris–HCl (pH 7.5), 1 mM DTT, and 0.1 mg/ml BSA]. The incubations were performed on ice for 30 min (for RNA) or at 37°C for 15 min (for DNA and nucleosomes). After incubation, 1 μl of 30% sucrose was added, and the samples were loaded onto 5% native polyacrylamide gels in 0.5× Tris–borate–EDTA. Gels were run at 100 V and 4°C (for RNA) or at room temperature (for DNA and nucleosomes). Nucleic acids were stained with SYBR Gold (Thermo Fisher Scientific) and visualized using ChemiDoc Imaging System (Bio-Rad, Hercules, CA, USA). The unbound fraction was quantified using ImageLab (Bio-Rad). The percentage of the bound fraction was obtained by subtracting the unbound value from the value with no protein added. Curve fitting was performed against bound fraction using Igor Pro software (WaveMetrics, Portland, OR, USA).

### Coimmunoprecipitation

Exponentially growing cells (1 × 10^9^ cells) were treated with ice-cold stop buffer (150 mM NaCl, 50 mM NaF, 10 mM EDTA, 1 mM NaN_3_). After vortexing and centrifugation at 2430 × *g* for 10 min at 4°C, cells were resuspended with lysis buffer [50 mM HEPES/NaOH (pH7.9), 150 mM NaCl, 1 mM EDTA, 1% NP40, 50 mM Na_3_VO_4_, 1 mM DTT, 1 mM PMSF, 1× Complete EDTA free (Roche Life Science)] and disrupted by glass-bead breakage in a Multi Beads Shocker (Yasui Kikai, Osaka, Japan). The crude lysate was centrifuged, and the supernatants were immunoprecipitated with anti-Flag antibody (A8592; Sigma) and protein A-conjugated magnetic beads (Dynabeads; Thermo Fisher Scientific) for 2 h at 4°C. After immunoprecipitation, the beads were washed twice with lysis buffer. The bound proteins were eluted with SDS sample buffer, boiled at 37°C or 95°C for 3 min, and analyzed by SDS–PAGE, followed by western blotting.

### Cross-linking mass spectrometry

Purified Clr4 (5 nmol in total) was incubated with BS3 (90 μM; Dojindo, Kamimashiki, Japan) in reaction buffer [10 mM HEPES–NaOH (pH 7.5), 50 mM NaCl] for 30 min at room temperature. The cross-linking reaction was stopped by adding Tris–HCl (pH 7.5) at 100 mM. The cross-linked protein was concentrated in Amicon Ultra Spin Concentrators and fractionated by gel filtration chromatography in gel filtration buffer [Superose6 10/300 GL, Cytiva; 20 mM HEPES–NaOH (pH 8.0), 300 mM NaCl]. A portion of the peak fractions was analyzed by SDS–PAGE using 4%–20% gradient gel (CosmoBio, Tokyo, Japan) and silver staining (SilverQuest Silver Staining Kit; Thermo Fisher Scientific). Fractions containing cross-linked Clr4 were concentrated in Amicon Ultra Spin Concentrators. Cross-linked proteins were treated with TCEP/IAA, and alkylated and digested by trypsin. The peptides were fractionated by GL-Tip SDB-SCX (GL Sciences, Tokyo, Japan) and loaded into a mass spectrometer (Orbitrap; Thermo Fisher Scientific). MS/MS spectra were analyzed using XlinkX. The data were visualized using xiNET [40].

### Silencing assay

Silencing assays were performed using unsaturated cultures grown in YEA (yeast extract with adenine) medium. Cells were harvested by centrifugation and resuspended in water. Serial 10-fold dilutions were spotted on plates with nonselective medium, yeast extract without adenine, minimal medium without uracil (–Ura), and minimal medium with 5-fluoroorotic acid. The plates were incubated at 30°C for 3−4 days.

### Chromatin immunoprecipitation

ChIP analyses were performed as described previously [41], with some modifications. Cells were grown at 30°C in YEA until the cell density reached 1 × 10^7^ cells/ml and fixed with 1% formaldehyde for 20 min at 25°C. After the fixation was stopped by addition of 125 mM glycine, the fixed cells were harvested by centrifugation at 771 × *g* for 2 min at 4°C, washed twice with ice-cold PBS, frozen in liquid nitrogen, and stored at −80°C until further use. Antibodies were preincubated with mouse IgG- or Protein G-conjugated magnetic beads (Dynabeads; Thermo Fisher Scientific). Cells were lysed with ChIP lysis buffer [50 mM HEPES–KOH (pH 7.5), 140 mM NaCl, 1 mM EDTA, 1% Triton-X, 0.5% Na-deoxycholate, 1 mM PMSF, 1× Complete EDTA-free (Roche Life Science)] and disrupted by glass-bead breakage in a Multi Beads Shocker (Yasui Kikai). Chromatin was sheared by sonication using Bioruptor (CosmoBio) and incubated with antibody-bound magnetic beads at 4°C overnight. The beads were washed twice with ChIP lysis buffer, twice with ChIP lysis buffer containing 0.5 M NaCl, twice with wash buffer [10 mM Tris–HCl (pH 8.0), 250 mM LiCl, 1 mM EDTA, 0.5% NP-40, 0.1% Na-deoxycholate], and twice with TE buffer [10 mM Tris–HCl (pH 8.0) and 1 mM EDTA]. Precipitated chromatin was incubated with RNase A (Thermo Fisher Scientific), treated with proteinase K (Sigma–Aldrich), and reverse cross-linked in reverse cross-linking buffer [20 mM Tris–HCl (pH 8.0), 1 mM EDTA, 0.8% SDS]. DNA samples were purified by NucleoSpin gel and PCR Clean-up (Macherey-Nagel, Düren, Germany). Quantitative PCR (qPCR) was performed using Luna qPCR Master Mix (NEB) and a real-time PCR machine [StepOnePlus™; Applied biosystems (ABI), Thermo Fisher Scientific]. The primers used in the ChIP analyses are described in Supplementary Table S3.

### Reverse transcription quantitative PCR (RT-qPCR)

RNA was extracted as follows: exponentially growing cells (∼1 × 10^8^ cells) were harvested and resuspended in TES buffer [10 mM Tris–HCl (pH 7.5), 10 mM EDTA, 0.5% SDS]. The cells were vortexed with an equal volume of phenol/chloroform (pH 5.2) and incubated at 65°C for 1 h. The mixture was chilled on ice and centrifuged. The aqueous phase was collected, vortexed with an equal volume of phenol/chloroform (pH 5.2), and centrifuged again. The aqueous phase was collected and precipitated with sodium acetate (pH 5.2) and ice-cold ethanol. After centrifugation, the supernatant was removed and the pellet was dried and resuspended in RNase-free water. After DNase I treatment, the resuspension was vortexed with an equal volume of phenol/chloroform (pH 5.2) and precipitated again with ethanol and sodium acetate. After centrifugation, the supernatant was removed and the pellet was dried and resuspended in RNase-free water. qPCR was performed using Luna Universal One-Step RT-qPCR Kit (NEB) and a real-time PCR machine (StepOnePlus™, ABI; Thermo Fisher Scientific). The primers used in the ChIP analyses are described in Supplementary Table S3.

### Immunoblotting

Immunoblotting was performed as described previously [41], with some modifications. Exponentially growing cells (1–2 × 10^8^ cells) were harvested and resuspended in alkaline 2-ME solution (1.85 N NaOH and 1.07 M 2-mercaptoethanol), and incubated on ice for 5 min. The cell suspension was mixed with 50% trichloroacetic acid and incubated for 5 min on ice. Cellular proteins were precipitated by centrifugation and the protein pellets were resuspended in SDS sample buffer, followed by boiling at 95°C for 3 min. Protein samples were analyzed using SDS–PAGE and subjected to western blotting.

### Microscopy

Yeast cells overexpressing EGFP-fused Clr4 were grown at 30°C in minimal medium without leucine until the cell density reached 1–2 × 10^7^ cells/ml. The cells were washed twice with water and incubated with Hoechst 33342 solution for 15 min at room temperature. The Hoechst solution was removed by centrifugation and the cells were resuspended in minimal medium without leucine. The cell suspension was mounted on coverslips. All images were captured using softWoRx software with CoolSNAP HQ^2^ camera (Photometrics; Tucson, AZ, USA) mounted on a DeltaVision Elite microscope (Cytiva) equipped with an Olympus PlanApo 100x objective lens (Evident; Tokyo, Japan). The set of Z-sections was taken at 10 focal planes with 0.3 μm intervals. All images were analyzed using ImageJ or Photoshop.

### Peptide pull-down assays

Peptide pull-down assays were performed as previously described [42], with some modifications. The peptides used in this study were unmodified H3 [H3unmod (ARTKQTARKSTGGKAPRKQL-C)] and K9-trimethylated H3 [H3K9me3 (ARTKQTARK_me3_STGGKAPRKQL-C)] (GenScript). The peptides (250 nmol) were covalently linked to 250 μl of SulfoLink Sepharose Resin (Thermo Fisher Scientific) via an artificially added C-terminal cysteine residue. Recombinant Clr4 (0.5 mmol) was incubated with 10 μl beads in 150 μl of binding buffer [20 mM Tris–HCl (pH 8.0), 150 mM NaCl, 1 mM EDTA, 0.1%, Triton X-100] for 1 h at room temperature. The beads were washed twice with 500 μl of binding buffer. The bound proteins were eluted with 50 μl of 2× SDS buffer with boiling at 95°C for 3 min. The protein samples were analyzed using SDS–PAGE and CBB staining.

### RNA immunoprecipitation

RNA-IP was performed as described previously [25, 38], with some modifications. Exponentially growing cells (2 × 10^9^ cells) were fixed with 1% formaldehyde for 30 min at 25°C. After the fixation was stopped by adding 125 mM glycine, the fixed cells were harvested by centrifugation at 771 × *g* for 2 min at 4°C and washed twice with ice-cold PBS. Antibodies were preincubated with protein G-conjugated magnetic beads (Dynabeads; Thermo Fisher Scientific). Cells were lysed with RIP lysis buffer [50 mM HEPES–KOH (pH 7.5), 140 mM NaCl, 1 mM EDTA, 1% Triton X-100, 0.5% sodium deoxycholate, 1 mM PMSF, 1× Complete EDTA-free (Roche Life Science), 0.01 U/μl RNase inhibitor (RNaseOUT; Thermo Fisher Scientific)] and disrupted by glass-bead breakage in a Multi Beads Shocker (Yasui Kikai). Chromatin was sheared by sonication using Bioruptor (CosmoBio) and incubated with antibody-bound magnetic beads at 4°C for 4 h. The beads were washed three times with RIP lysis buffer and twice with TE buffer [10 mM Tris–HCl (pH 8.0), 1 mM EDTA, 0.01 U/μl RNase inhibitor]. Precipitated RNA was treated with proteinase K and reverse cross-linked in proK buffer [20 mM Tris–HCl (pH 8.0) and 1 mM EDTA, 0.8% SDS, 2 mg/ml proteinase K (Sigma–Aldrich), 0.2 U/μl RNase inhibitor]. Each sample was extracted three times with phenol/chloroform isoamyl alcohol (pH 5.2), precipitated with ice-cold ethanol containing sodium acetate (pH 5.2) and glycogen, and treated with 5 U of DNase I (Takara Bio). To stop DNase I digestion, 2.5 μM EDTA was added to the samples, followed by heating at 80°C for 10 min. After ethanol precipitation (as described above), RNAs were resuspended in RNase-free water. qPCR was performed using Luna Universal One-Step RT-qPCR Kit (NEB) and a real-time PCR machine (StepOnePlus™, ABI; Thermo Fisher Scientific). The primers used in the RNA-IP analyses are described in Supplementary Table S3.

## Results

### N-terminus of Clr4 interacts with its C-terminal catalytic domain *in vitro*

**Figure 1.**
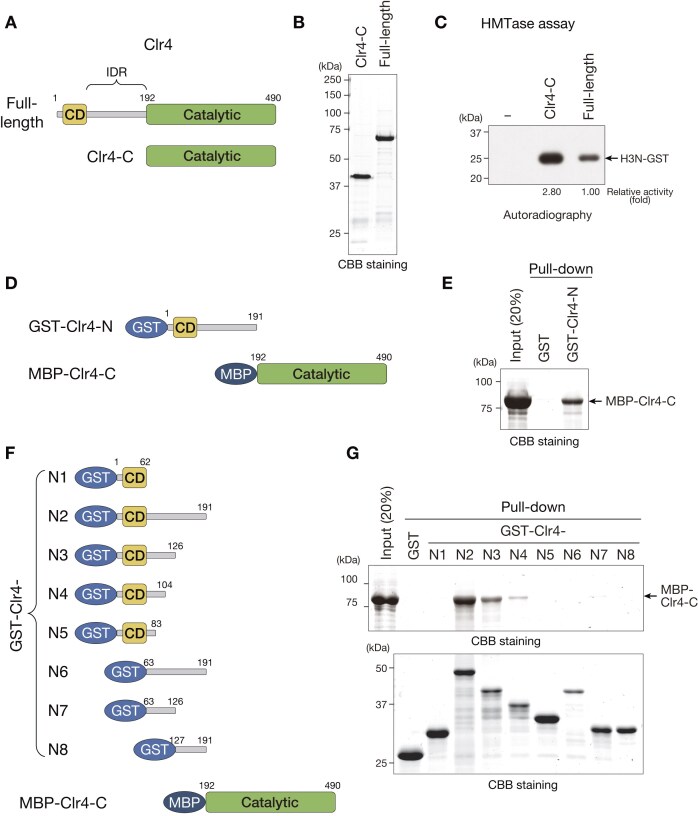
N-terminus of Clr4 interacts with its C-terminal catalytic domain *in vitro*. (**A**) Schematic diagrams of full-length or N-terminally deleted (Clr4-C) Clr4. CD, chromodomain; IDR, intrinsically disordered region. (**B**) Recombinant Clr4 proteins used in panel (C) were resolved by 8% SDS–PAGE and visualized by CBB staining. (**C**) Representative image of *in vitro* HMTase assays using recombinant Clr4 proteins. H3N-GST was used as the substrate. The signals of ^3^H-labeled H3N-GST were calculated, and relative fold activities are shown beneath each lane. (**D**) Schematic diagrams of GST-fused Clr4 N-terminus (GST-Clr4-N) and MBP-fused catalytic domain of Clr4 (MBP-Clr4-C). (**E**) Representative image of GST pull-down assays using recombinant Clr4 proteins. (**F**) Schematic diagrams of GST-fused Clr4 N-terminus with N-terminally or C-terminally deletion (GST-Clr4-N1∼N8) and MBP-Clr4-C. (**G**) Representative image of GST pull-down assays using recombinant Clr4 proteins.

We previously showed that Clr4 lacking the CD and IDR exhibits increased H3K9 methyltransferase activity *in vitro* [32]. To investigate the molecular details of this effect, we first verified our previous observations. We prepared recombinant proteins of full-length Clr4 and its C-terminal catalytic domain (Clr4-C, amino acids 192−490) and performed *in vitro* HMTase assays. In this assay, H3N-GST was used as a substrate, and the incorporation of a radiolabeled methyl group transferred from ^3^H-SAM was detected by autoradiography (Fig. 1A–C). Consistent with the results of previous studies [32], Clr4-C exhibited higher activity than full-length Clr4, supporting the notion that the N-terminal region of Clr4 inhibits the enzymatic activity of its C-terminal catalytic domain.

To determine whether the Clr4 N-terminus physically interacts with the C-terminal catalytic domain, we prepared the Clr4 N-terminus comprising the CD and IDR (Clr4-N, amino acids 1−191) and the Clr4-C as GST- and MBP-fused proteins, respectively (Fig. 1D), and performed a GST pull-down assay. This assay revealed that GST-Clr4-N interacted with the MBP-Clr4-C (Fig. 1E). To identify the regions involved in this interaction, we prepared a series of Clr4 N-terminal fragments as GST-fused proteins (GST-Clr4-N1–N8; Fig. 1F) and examined their ability to interact with the MBP-Clr4-C. We found that neither the CD alone (N1) nor the IDR alone (N6–N8) interacted with the MBP-Clr4-C (Fig. 1G), suggesting that both the CD and IDR are required for interaction. In addition, the interaction between GST-Clr4-N and MBP-Clr4-C gradually weakened when the IDR of Clr4-N was deleted (N2–N5), suggesting that the CD and several regions of the IDR contribute to the stable interaction between Clr4-N and Clr4-C.

### Basic residues in the Clr4 IDR are involved in the interaction with the C-terminal catalytic domain and autoinhibition of its enzymatic activity

To determine the regions involved in the interaction between the N-terminus and the C-terminal catalytic domain of Clr4, we performed cross-linking mass spectrometry (XL-MS) (Supplementary Fig. S1). Full-length Clr4 was produced as a recombinant protein and cross-linked with BS3 (Supplementary Fig. S1A and B). Cross-linked Clr4 was separated by gel filtration chromatography, followed by trypsin digestion and MS analysis (Supplementary Fig. S1B and C). Because the migration patterns of cross-linked Clr4 differed between early (fractions 32–34) and late (fractions 35–37) eluting fractions, these fractions were pooled separately and subjected to MS analysis (Supplementary Fig. S1C). This analysis revealed multiple interactions between the N-terminus and the C-terminal catalytic domain as well as within the N-terminus (Supplementary Tables S1 and S2), and the positions of the cross-linked residues were overlapped significantly between experiments using early- and late-eluting fractions (Supplementary Fig. S1D and E). Focusing on the interactions between the N-terminus and C-terminal catalytic domain (Supplementary Fig. S1D and E, indicated by red arcs), three regions in the N-terminus, a region near the N-terminus of the CD, a region near the center of the IDR, and a region near the C-terminus of the IDR were mainly cross-linked to the C-terminal catalytic domain (Supplementary Fig. S1D and E, indicated by red bars). Interestingly, the cross-linked residues in the C-terminal catalytic domain were concentrated in two regions: the N-terminal region of the SET domain and near the C-terminal end corresponding to the ARL (Supplementary Fig. S1D and E, indicated by red bars). Since BS3 cross-links the primary amine of lysine residues, it is likely that cross-linked lysine residues or residues near them are involved in the interaction.

To investigate the importance of the residues identified by XL-MS in the interaction, we first focused on the ARL in the C-terminal catalytic domain (Supplementary Fig. S2A). The ARL autoinhibits Clr4 methyltransferase activity by excluding H3 from the catalytic pocket, and this autoinhibition is abolished by automethylation of lysine residues within the ARL [31]. We introduced several amino acid substitutions within or adjacent to the ARL of MBP-Clr4-C, and examined the interaction with GST-Clr4-N by pull-down assay. We found that both the construct containing lysine-to-alanine substitutions of two lysine residues targeted for automethylation (K2A) and that containing additional alanine substitutions of two basic residues in the ARL (K4A) did not alter, but rather slightly increased, the interaction with GST-Clr4-N (Supplementary Fig. S2A and B). Furthermore, four glutamine-to-alanine substitutions in the ARL (Q4A), deletion of the central region of the ARL (Δ), replacement of the Clr4 ARL with the corresponding region of DIM5, the Clr4 homologue from *Neurospora crassa*, or replacement of the ARL with a glycine–serine linker did not significantly reduce the interaction (Supplementary Fig. S2A and B). In addition, replacement of a basic amino acid block, KLRR, at the C-terminal junction of the ARL with alanine (472–475A) or glutamic acid (472–475E) did not alter the interaction (Supplementary Fig. S2A and C). These results suggest that amino acids within or adjacent to the ARL are not involved in the interaction between the N-terminus of Clr4 and the C-terminal catalytic domain.

Next, we focused on the IDR, which contains amino acid residues cross-linked to the C-terminal catalytic domain. Because the interaction between GST-Clr4-N and MBP-Clr4-C was enhanced in a low salt condition (0 mM NaCl) or abolished in a high salt condition (500 mM NaCl), electrostatic interactions involving charged residues appeared to be involved in the interaction (Supplementary Fig. S2C). The IDR between the CD and the C-terminal catalytic domain contains 129 amino acids, ∼30% of which are charged residues, either basic or acidic (Supplementary Fig. S3A), and cross-linked lysine residues in the XL-MS are distributed throughout the IDR (Supplementary Fig. S3A, indicated by asterisks). We divided the IDR into six blocks (blocks A–F)—introduced amino acid substitutions into GST-Clr4-N, in which the charged residues in each block or in the combined several different blocks were substituted by serine, uncharged amino acid—and examined their interaction with MBP-Clr4-C by pull-down assay. We found that GST-Clr4-N containing amino acid substitutions in blocks A, D, and F (Mut ADF) slightly attenuated the interaction with MBP-Clr4-C, whereas amino acid substitutions in either block B (Mut B) or C (Mut C) significantly decreased the interaction between GST-Clr4-N and MBP-Clr4-C (Supplementary Fig. S3B). Notably, combined amino acid substitutions in both blocks B and C (Mut BC) almost completely abolished the interaction (Supplementary Fig. S3B), suggesting that charged residues in blocks B and C play a critical role in the interaction with the C-terminal catalytic domain.

**Figure 2.**
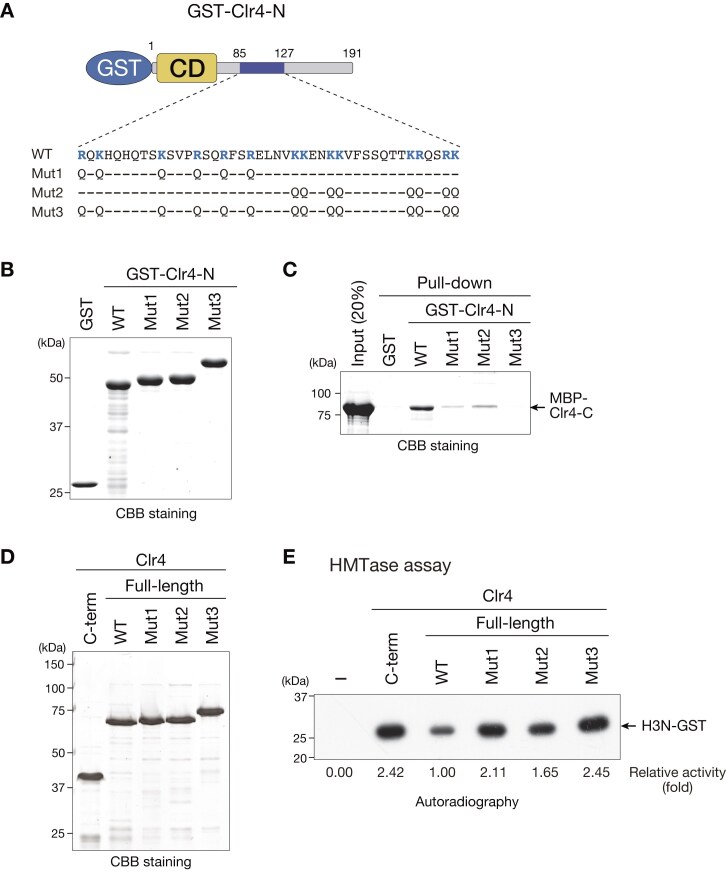
Basic residues in the Clr4 IDR are involved in the interaction with the C-terminal catalytic domain and autoinhibition of its enzymatic activity. (**A**) A schematic diagram of GST-Clr4-N with alignment of amino acid sequences of wild type and three mutants (Mut1–3). Basic amino acid residues are shown in blue. (**B**) Recombinant proteins used in panel (C) were resolved by 10% SDS–PAGE and visualized by CBB staining. (**C**) Results of GST pull-down assays using recombinant Clr4 proteins. (**D**) Recombinant proteins used in panel (E) were resolved by 8% SDS–PAGE and visualized by CBB staining. (**E**) Representative images of *in vitro* HMTase assays using H3N-GST as the substrate. The signals of ^3^H-labeled H3N-GST were calculated and relative fold activities are shown beneath each lane.

In blocks B and C, most of the charged residues are lysine and arginine (Supplementary Fig. S3A), and to further confirm their involvement in the interaction with the C-terminal catalytic domain, we substituted these basic residues with glutamine, an uncharged amino acid with a similar side chain (Fig. 2A). We then prepared wild-type GST-Clr4-N and three mutants containing Mut1, K/R > Q substitutions in block B; Mut2, K/R > Q substitutions in block C; or Mut3, K/R > Q substitutions in both blocks B and C (Fig. 2B) and examined their interaction with MBP-Clr4-C by pull-down assays (Fig. 2C). As observed for the mutants with serine substitutions (Supplementary Fig. S3B), Mut1–3 reduced the interaction with MBP-Clr4-C (Fig. 2C). Although a weak interaction was still detected for mutants with Mut1 or Mut2, Mut3 showed the strongest effect and almost completely abolished the interaction with MBP-Clr4-C, suggesting that the basic residues in blocks B and C work cooperatively to interact with the C-terminal catalytic domain. As acidic residues are concentrated at the H3-tail binding site in the C-terminal catalytic domain [33], the basic residues in the blocks B and C may be involved in the interaction with these acidic residues.

To investigate the effect of the interaction between the N-terminus and C-terminal catalytic domain of Clr4 on its HMTase activity, we prepared full-length, wild-type, and mutant Clr4 containing Mut1, Mut2, or Mut3 (Fig. 2D) and used them for *in vitro* HMTase assay as in Fig. 1C. In this assay, Clr4-C was used as a control. We found that all Clr4 mutants (Mut 1–3) exhibited higher HMTase activity against H3N-GST than wild-type Clr4 (Fig. 2E). Notably, their activities showed an inverse correlation with the pull-down assay (Fig. 2C), and more importantly, the activity of Clr4-Mut3 was comparable to that of Clr4-C (Fig. 2E). A luminescence-based HMTase assay confirmed that the mutant Clr4 exhibited higher activity against H3 peptides than the wild-type Clr4 (Supplementary Fig. S4). These results support the notion that the enzymatic activity of Clr4 is autoinhibited by the interaction between the IDR and C-terminal catalytic domain, and that basic residues in blocks B and C play a critical role in this autoinhibition.

### Basic residues in the Clr4 IDR play an important role in its function of heterochromatic gene silencing

**Figure 3.**
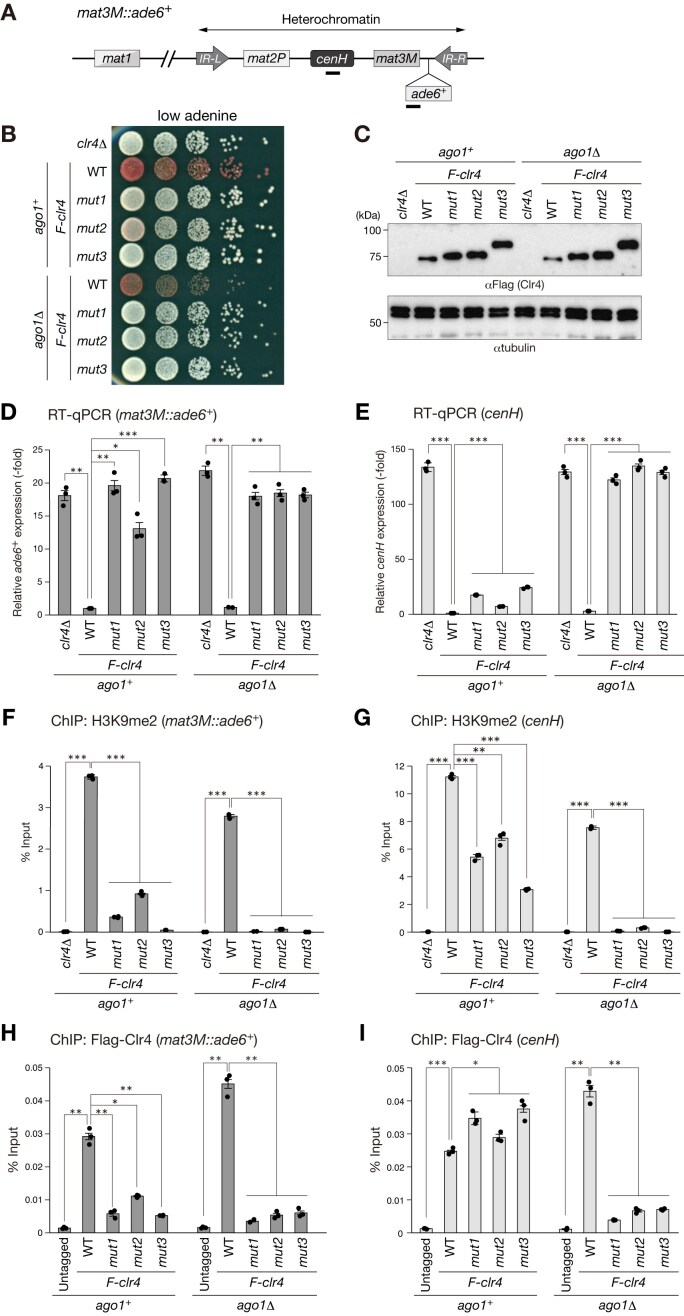
Basic residues in the IDR contribute to the function of Clr4 in heterochromatin spreading. (**A**) Schematic diagram of the mating-type locus showing the *mat3M::ade6*^+^ insertion site. Black bars indicate the target regions of RT-qPCR and ChIP-qPCR for *mat3M::ade6*^+^ and *cenH*. (**B**) Heterochromatic silencing assays of control cells lacking *clr4* (*clr4*Δ) and cells expressing FLAG-tagged wild-type (*F-clr4^WT^*) or mutant Clr4 (*F-clr4^mut1^*, *F-clr4^mut2^*, *F-clr4^mut3^*) both with and without *ago1*^+^ deletion (*ago1*Δ). Silencing at *mat3M::ade6*^+^ was evaluated. Ten-fold serial dilutions of the indicated strains were spotted onto adenine-limited medium. (**C**) Whole cell lysates prepared from the yeast strains used in panel (B) were subjected to immunoblotting using the indicated antibodies. Levels of *mat3M::ade6*^+^ (**D**) and *cenH* (**E**) transcripts were quantified by RT-qPCR, relative to the control cell (*F-clr4^WT^*). ChIP analysis of H3K9me2 levels at *mat3M::ade6*^+^ (**F**) and *cenH* locus (**G**), relative to the control *act1*^+^. ChIP analysis of Flag (Clr4) levels at *mat3M::ade6*^+^ (**H**) and *cenH* locus (**I**), relative to the control *act1*^+^. In panels (D)–(I), statistical significance was determined using a two-tailed unpaired Student’s *t*-test. *P< .05, **P< .005, ***P< .0005. Error bars: standard deviation (SD); *n* = 3.

Having established the role of the Clr4 IDR in autoregulation *in vitro*, we next determined the biological effect of the interaction between the IDR and the C-terminal catalytic domain of Clr4 on heterochromatin assembly *in vivo*. We generated yeast strains expressing N-terminal FLAG-tagged, wild-type, or mutant (Mut1–3) Clr4 from its endogenous locus and performed silencing assays (Fig. 3A–C). These strains also carry a marker gene inserted near the *mat3-M* locus (*mat3M::ade6*^+^), which is normally silenced by heterochromatin spreading (Fig. 3A). Strains expressing wild-type Clr4 formed red colonies on adenine-limited media, whereas the *clr4* deletion strain (*clr4*Δ) formed white colonies, as heterochromatin was abolished, and the marker gene was derepressed (Fig. 3B). Interestingly, we found that cells expressing mutant Clr4 (Mut1–3) formed white or pink colonies on adenine-limited media, indicating that these mutations cause a defect in silencing at the *mat3M* locus (Fig. 3B; *ago1*^+^). The mutant Clr4 proteins were stably expressed in yeast cells (Fig. 3C), and their mobility in the SDS–PAGE gel decreased, presumably due to a reduction in positively charged residues (Fig. 3C). Additionally, the mutant protein levels appeared higher than those of wild-type Clr4 (Fig. 3C). The reason for this effect is unclear, but the residues in the hinge region may determine the Clr4’s cellular stability. Given that mutant Clr4 (Mut1–3) exhibited a higher HMTase activity than wild-type Clr4 *in vitro* (Fig. 2E and Supplementary Fig. S4), these results were unexpected and contrary to our prediction that mutant cells would promote robust heterochromatin formation.

The defect in heterochromatin assembly at the *mat* locus in *clr4* mutant strains could be due to amino acid substitutions in the N-terminal IDR of Clr4 that affect its association with the CLRC or its nuclear localization. To test the first possibility, we generated strains expressing both FLAG-tagged, wild-type, or mutant Clr4 and Myc-tagged Rik1, one of the core components of the CLRC, and performed a coimmunoprecipitation assay to examine their interactions [43]. This assay revealed that mutant Clr4 could stably interact with Rik1 with an affinity comparable to that of wild-type Clr4 (Supplementary Fig. S5A), suggesting that amino acid substitutions in the IDR have a negligible effect on its association with the CLRC. To test the second possibility, we expressed EGFP-fused, wild-type or mutant Clr4 and examined their cellular localization. The results showed that, although a small population of Clr4^Mut3^ was detected in the cytoplasm, all Clr4 mutants were predominantly localized to the nucleus and formed foci that likely corresponded to heterochromatic regions (Supplementary Fig. S5B). These results suggest that amino acid substitutions in the N-terminal IDR of Clr4 have negligible effects on its association with CLRC or its nuclear localization and that the basic residues in the Clr4 IDR play additional role(s) other than autoinhibition *in vivo*.

### Basic residues in the IDR contribute to the function of Clr4 in heterochromatin spreading

To further investigate why amino acid substitutions in the Clr4 IDR result in a silencing defect, we analyzed the heterochromatin state in detail by performing RT-qPCR and ChIP-qPCR (Fig. 3D–I). Consistent with the silencing assay (Fig. 3B), higher levels of *mat3M::ade6*^+^ expression were detected in the *clr4* mutant strains, and the levels were comparable to those of *clr4*Δ cells (Fig. 3D, *ago1*^+^). Similarly, H3K9me2 was localized to the *mat3M::ade6*^+^ locus in wild-type cells, whereas the amino acid substitutions in the IDR (Mut1–3) strongly reduced H3K9me2 at the *mat3M::ade6*^+^ locus (Fig. 3F, *ago1*^+^). The reduced levels of H3K9me2 in the mutant cells were accompanied by the reduction of Clr4 associated with the *mat3M::ade6*^+^ locus (Fig. 3H, *ago1*^+^). Taken together, these results suggest that the basic residues in the Clr4 IDR play an important role in its chromatin association to induce H3K9me2 at the *mat3M::ade6*^+^ locus.

It is generally accepted that euchromatic genes inserted into heterochromatic regions are silenced by spreading of heterochromatic H3K9me and associated machinery from adjacent regions containing the heterochromatin nucleation sites [44]. At the *mat* locus, the centromere homologous repeat, *cenH*, functions as a heterochromatin nucleation center by recruiting the RITS complex, and the RITS complex itself and H3K9me spread from the *cenH* region throughout the *mat* locus (Fig. 3A) [15, 45]. Interestingly, when we analyzed the heterochromatin state of the *cenH* region, we found that *cenH* expression was derepressed in *clr4*Δ cells but maintained at low levels in *clr4* mutant cells (Fig. 3E, *ago1*^+^), and that although reduced, substantial levels of H3K9me2 were retained at the *cenH* region in *clr4* mutant cells (Fig. 3G, *ago1^+^*). In addition, mutant Clr4 stably associated with the *cenH* region at levels comparable to those of wild-type Clr4 (Fig. 3I, *ago1*^+^). Taken together, these results suggest that the basic residues in the IDR specifically contribute to Clr4 function in spreading H3K9me to the inserted *mat3M::ade6*^+^ region.

To verify that the role of the basic residues in the IDR in spreading H3K9me is not specific to *mat3M::ade6*^+^, the effect of *clr4* mutations on *Kint2::ura4*^+^ silencing, in which an *ura4^+^* marker gene is inserted into the *cenH* proximal region, was also examined (Supplementary Fig. S6A). As observed for *mat3M::ade6^+^*, the *clr4* mutant strains showed a silencing defect (Supplementary Fig. S6B), derepression of *Kint2::ura4^+^* (Supplementary Fig. S6C), and reduced H3K9me2 levels at the *Kint2::ura4^+^* region (Supplementary Fig. S6E), whereas *cenH* expression remained silenced (Supplementary Fig. S6D), and wild-type levels of H3K9me2 were present at the *cenH* region (Supplementary Fig. S6F). These results strengthen our conclusion that basic residues in the IDR contribute to the function of Clr4 in heterochromatin spreading.

Heterochromatin spreading is closely related to the H3K9me read–write function of Clr4, which is regulated by the binding of its CD to H3K9me. To investigate the effect of the IDR mutation on the CD’s H3K9me-binding activity, we performed peptide pull-down assays. The mutant Clr4 bound H3K9me3 peptides similarly to the wild-type Clr4, whereas neither the wild-type nor the mutant Clr4 bound H3K9-unmodified peptides (Supplementary Fig. S7). These results suggest that the spreading defect observed in *clr4* mutants is not caused by a defect in the CD’s H3K9me binding activity.

### Role of the Clr4 IDR in heterochromatin spreading is linked to RNAi

Heterochromatin assembly and spreading are mediated by the cooperative action of several mechanisms. In addition to the read–write mechanism involving the CD and catalytic domain of Clr4, the coordinated action of the RNAi and Clr4 is important for heterochromatin spreading. Although the *cenH* recruits the RITS complex and functions as a heterochromatin nucleation center, the spreading of RITS throughout the *mat* locus requires H3K9me by Clr4 [45]. In contrast, in RNAi mutant cells, H3K9me is maintained at the pericentromeric repeat regions, but is lost at an inserted marker gene [43]. To explore the functional relationship between the Clr4 IDR and RNAi pathway, we combined the *clr4* mutations (*mut1–3*) with *ago1*Δ and examined the effect on heterochromatin assembly (Fig. 3B and C). Deletion of *ago1* alone had almost no effect on *mat3M::ade6^+^* silencing (Fig. 3B, *ago1*Δ *F-clr4*^WT^), as an RNAi-independent mechanism also contributes to heterochromatin assembly at the *mat* locus [27]. However, when the *ago1* deletion was combined with any of the *clr4* mutations (*mut1–3*), double mutants exhibited a severe silencing defect, and as judged by colony color, their defect appeared to be more severe than that of mutants with any of the *clr4* mutations (*mut1–3*) alone (Fig. 3B). As observed for *clr4* mutants in *ago1*^+^ background, *mat3M::ade6^+^* expression was increased to levels comparable to those of *clr4*Δ (Fig. 3D), and the levels of H3K9me2 and associated Clr4 at the *mat3M::ade6^+^* region were reduced in double mutants with *ago1*Δ (Fig. 3F and H). Interestingly, although *cenH* expression was maintained at low levels in *clr4* mutants or *ago1*Δ cells, it was markedly increased in double mutants (Fig. 3E). Similarly, substantial levels of H3K9me2 were detected at the *cenH* locus in *clr4* mutant or *ago1*Δ cells, but were completely lost in double mutants (Fig. 3G), which was accompanied by the loss of mutant Clr4 associated with the *cenH* locus (Fig. 3I). Taken together, these results suggest that the Clr4 IDR functionally cooperates with the RNAi pathway to assemble heterochromatin at the *cenH* locus.

The functional link between the Clr4 IDR and RNAi pathway was also examined at pericentromeric heterochromatin. At pericentromeric repeat regions, the RNAi pathway plays a primary role in heterochromatin assembly, and in RNAi mutants, H3K9me is lost at the inserted marker regions, but is partially maintained at centromeric *dg* and *dh* repeat regions [24, 43]. Silencing assays and RT-qPCR analyses revealed that cells expressing mutant Clr4 exhibited a weak silencing defect at the *otr1R::ade6^+^* locus (Supplementary Fig. S8B and C). Deletion of *ago1* completely abolished *otr1R::ade6^+^* silencing and H3K9me at the *otr1R::ade6^+^* locus, and the combination of *clr4* mutations (Mut1–3) with *ago1*Δ had no additive effect on *otr1R::ade6^+^* silencing or H3K9me at the *otr1R::ade6^+^* locus (Supplementary Fig. S8C and E). Consistent with previous studies [43], H3K9me2 was retained at the *dg* repeat region in *ago1*Δ cells (Supplementary Fig. S8F, *ago1*Δ *F-clr4^WT^*), but was lost when *ago1* deletion was combined with *clr4* mutations (*mut1–3*) (Supplementary Fig. S8F, *ago1*Δ *F-clr4^mut1–3^*). Although the degree of contribution of RNAi and Clr4 to heterochromatin assembly differed between the *mat* locus and pericentromeric regions, these results also support the notion that the Clr4 IDR and RNAi function cooperatively in heterochromatin spreading.

At the *mat* locus, two ATF/CREB family proteins, Atf1 and Pcr1, function in a mechanism that parallels to the RNAi pathway for heterochromatin nucleation. Combining *ago1*Δ with either *atf1*Δ or *pcr1*Δ results in a defect in heterochromatic gene silencing [27]. To investigate the relationship between the Clr4 IDR and this mechanism, we examined the effect of combining *clr4* mutations (*mut1–3*) with *atf1*Δ or *pcr1*Δ on heterochromatin assembly. Deleting *atf1* or *pcr1* alone had a negligible effect on *mat3M::ade6^+^* silencing (Supplementary Fig. S9A), and H3K9me2 was localized to both the *mat3M::ade6*^+^ and *cenH* loci (Supplementary Fig. S9B–E). Combining the *atf1* or *pcr1* deletion with any of the *clr4* mutations (*mut1–3*) resulted in minor, additive effects on *mat3M::ade6*^+^ silencing and H3K9me2 levels (Supplementary Fig. S9A, B, and D). However, unlike *ago1*Δ cells (Fig. 3G), these double mutants did not cause obvious heterochromatin loss at the *cenH* locus (Supplementary Fig. S9C and E). These results suggest that neither Atf1 nor Pcr1 plays a cooperative role with the Clr4 IDR in the heterochromatin nucleation at the *cenH* locus.

### RNA binding of Clr4 IDR regulates its intramolecular interaction and enzymatic activity

Since the RNAi pathway is triggered by the transcription of heterochromatic repeats, Clr4 IDR function may also be involved in the association with transcribed RNAs. To gain further insight into the function of Clr4 IDR in heterochromatin spreading, we next investigated whether the basic residues in the Clr4 IDR are involved in RNA binding. Previously, we showed that the Clr4 CD has the ability to bind pericentromeric RNA [38], but the involvement of the IDR in Clr4 RNA binding has not been investigated. We prepared GST-fused Clr4-N and Clr4-C constructs (Fig. 1F) and performed EMSAs using pericentromeric RNA as a probe (Supplementary Fig. S10A and B). The GST-Clr4-N constructs containing the region within the Clr4 IDR containing blocks B and C (Supplementary Fig. S10A; constructs N2, N3, N6, and N7) efficiently bound to the pericentromeric RNA (Supplementary Fig. S10C and D). In contrast, the GST-Clr4-N8, which lacks blocks B and C, as well as the GST-Clr4-CD (N1) and GST-Clr4-C (C1) constructs, failed to bind to the pericentromeric RNA (Supplementary Fig. S10C and D).

**Figure 4.**
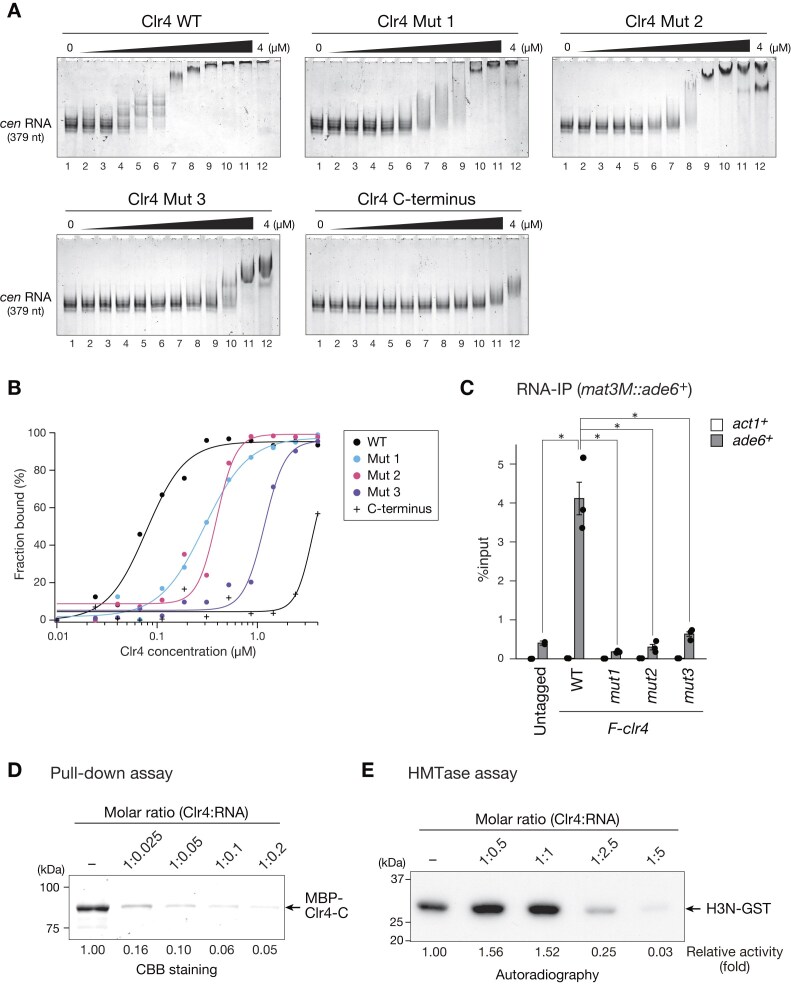
RNA binding of Clr4 IDR regulates its intramolecular interaction and enzymatic activity. (**A**) Representative results of EMSA using wild-type, mutant, or N-terminally deleted Clr4. 379 nt single-stranded RNA, corresponding to the pericentromeric repeat, was used as a probe. (**B**) Quantification of EMSA results in panel (A); the fraction of retarded RNA probe was plotted against each protein concentration. (**C**) RNA-IP experiments using yeast strains expressing wild-type or mutant Clr4. Immunoprecipitated RNA was subjected to RT-qPCR analysis using primers for the ade6 transcripts inserted into the mat locus (*mat3M::ade6*^+^). Statistical significance was determined using a two-tailed unpaired Student’s *t*-test. *P< .05. Error bars: SD; *n* = 2 or 3. (**D**) Representative image of GST pull-down assays using recombinant Clr4 proteins in the presence of the 379 nt single-stranded RNA corresponding to the pericentromeric repeat. The signals of pulled-down MBP-Clr4-C were calculated, and relative fold enrichments are shown beneath each lane. (**E**) Representative images of the *in vitro* HMTase assays using H3N-GST in the presence of the 379 nt single-stranded RNA corresponding to the pericentromeric repeat. The signals of ^3^H-labeled H3N-GST were calculated and relative fold activities are shown beneath each lane.

To investigate the role of basic residues in the blocks B and C of the Clr4 IDR in full-length Clr4’s RNA-binding activity, we prepared wild-type and mutant Clr4 and Clr4-C, and performed EMSAs using pericentromeric RNA as a probe. We found that Clr4 bound efficiently to the pericentromeric RNA and that mutant Clr4 (Mut1–3) exhibited a reduced RNA binding ability (Fig. 4A). Comparing the migration pattern of probes, the RNA binding activity was significantly reduced in Mut3 compared to that in Mut1 and Mut2 (Fig. 4A and B), suggesting that the basic amino acid residues in blocks B and C of the Clr4 IDR are cooperatively involved in the RNA binding of Clr4. To examine the sequence specificity of Clr4 IDR’s RNA binding, we performed EMSAs using RNA corresponding to the *ura4* gene (Supplementary Fig. S11). We found that Clr4 binds to *ura4* RNA with a comparable affinity to that of pericentromeric RNA, and mutant Clr4 (Mut1–3) exhibited reduced RNA binding, as observed for pericentromeric RNA (Supplementary Fig. S11). These results suggest that Clr4 binds to RNA without particular sequence specificity. To investigate the possible contributions of Clr4 IDR to its RNA binding *in vivo*, we performed RNA-IP assays. Wild-type Clr4 bound to RNA transcribed from the *mat3::ade6*^+^ locus, whereas the amount of associated RNA was significantly reduced for mutant Clr4 (Fig. 4C), suggesting that the basic residues in the Clr4 IDR are required for its RNA binding *in vivo*.

Since the basic residues of the Clr4 IDR are involved in the interaction with the C-terminal catalytic domain (Fig. 2C), we next investigated whether RNA affects the interaction between the IDR and C-terminal catalytic domain by performing GST pull-down assays in the presence of RNA. Interestingly, we found that the interaction between GST-Clr4-N and MBP-Clr4-C significantly decreased with the addition of RNA (Fig. 4D), suggesting that RNA interferes with the interaction between the IDR and C-terminal catalytic domain, and that RNA and the C-terminal catalytic domain compete to bind to the IDR. We next investigated whether RNA affects the HMTase activity of Clr4. HMTase assays were performed in the presence or absence of RNA. Notably, the HMTase activity of Clr4 was enhanced when RNA was added at a molar ratio of 1:0.5 or 1:1 (Fig. 4E). This is consistent with our results that mutant Clr4 defective in the interaction between the IDR and C-terminal catalytic domain exhibited higher HMTase activity (Fig. 2E). Moreover, the addition of excess RNA (at a molar ratio of 1:2.5 or 1:5) resulted in a reduction in HMTase activity (Fig. 4E). These results indicate that the autoinhibition and enzymatic activity of Clr4 are indeed regulated by RNA, but the effect is dependent on the amount of RNA present.

### Basic residues in the Clr4 IDR contribute to targeting of nucleosomal substrates

**Figure 5.**
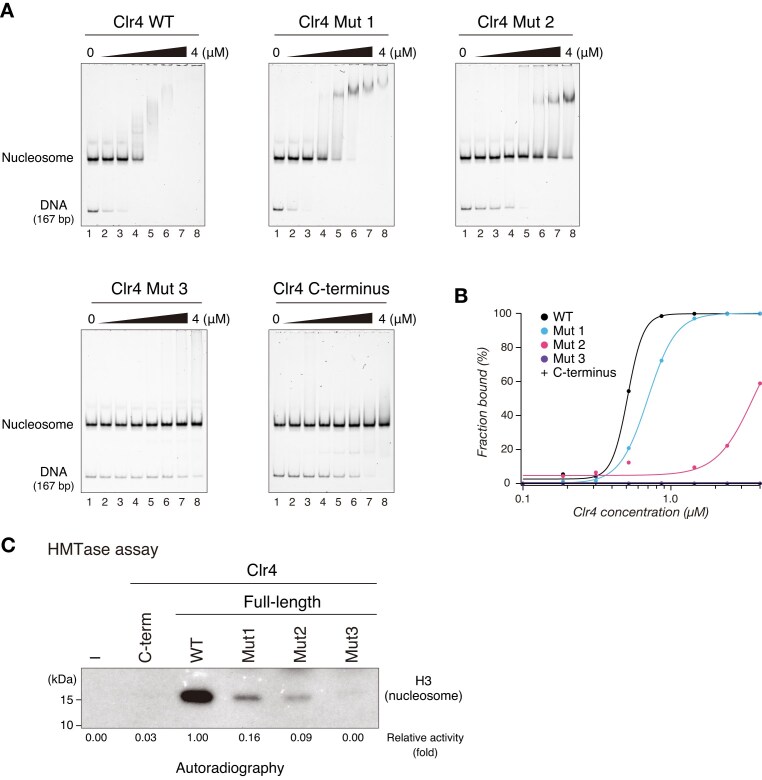
Basic residues in the Clr4 IDR contribute to targeting of nucleosomal substrates. (**A**) Representative results of EMSA using wild-type, mutant, or N-terminally deleted Clr4. Reconstituted mononucleosomes containing 167 bp DNA were used as the probe. (**B**) Quantification of EMSA results in panel (A); the fraction of retarded DNA probe was plotted against each protein concentration. (**C**) Representative images of *in vitro* HMTase assays using mononucleosomes as the substrate. The signals of ^3^H-labeled H3 in the nucleosomes were calculated, and relative fold activities are shown beneath each lane.

Allosteric activation of Clr4/Suv39h1 enzymatic activity is reportedly involved in heterochromatin spreading [19, 20]. In addition, the Clr4 IDR reportedly binds to nucleosomes [46]. To determine whether the basic residues of the Clr4 IDR contribute to targeting of nucleosomal substrates, we performed EMSAs using mononucleosomes as probes (Supplementary Fig. S12). The GST-Clr4-N constructs containing the region within the Clr4 IDR containing blocks B and C efficiently bound to the nucleosomes (Supplementary Fig. S12A–C; constructs N2, N3, N6, and N7). In contrast, the GST-Clr4-N8, GST-Clr4-CD (N1), and GST-Clr4-C (C1) constructs failed to bind to the nucleosomes (Supplementary Fig. S12C and D). Next, we examined the role of basic residues in blocks B and C of the Clr4 IDR in the nucleosome binding activity of the full-length Clr4 (Fig. 5). Wild-type Clr4 bound efficiently to the nucleosomes, whereas mutant Clr4 (Mut1 and Mut2) exhibited reduced binding activity compared to that of wild-type Clr4 (Fig. 5A and B). Importantly, Mut3 almost completely abolished the nucleosome binding activity of Clr4 (Fig. 5A and B). These results suggest that the basic residues of the Clr4 IDR are required for Clr4 binding to nucleosomal substrates and that the basic residues in blocks B and C of the Clr4 IDR function cooperatively in the nucleosome binding of Clr4. Although the nucleosome binding activity of Clr4 appeared to be mediated primarily by the interaction between the Clr4 IDR and DNA wrapped around core histones (Supplementary Figs S13 and S14), the effect of Clr4 mutations was more pronounced when nucleosomes were used as substrates than when DNA was used as a substrate (Fig. 5A and B). The interaction between Clr4 and nucleosomes may be modulated by unidentified interactions other than the interaction between the Clr4 IDR and nucleosomal DNA.

To explore the possible contribution of the basic residues of the Clr4 IDR to the methyltransferase activity of Clr4 against nucleosomes, we performed HMTase assays using mononucleosomes as substrates. Wild-type Clr4 efficiently methylated H3 in mononucleosomes (Fig. 5C). Interestingly, in contrast to the results when H3N-GST was used as substrate (Fig. 2E), mutant Clr4 (Mut1 and Mut2) exhibited reduced methyltransferase activity compared to wild-type Clr4, and Mut3 almost completely abolished Clr4 methyltransferase activity (Fig. 5C). Similarly, Clr4-C, which exhibited higher activity than full-length Clr4 against H3N-GST and H3 peptide (Fig. 2E and Supplementary Fig. S4), failed to methylate nucleosomal substrates (Fig. 5C). These results indicate that the basic residues in the Clr4 IDR play a critical role in the function of Clr4 to target and methylate chromatin substrates.

## Discussion

In *S. pombe*, the H3K9 methyltransferase Clr4 plays an essential role in heterochromatin assembly and gene silencing. Compared to that of the two conserved domains, CD and C-terminal catalytic domain, the role of the IDR in Clr4 function remains unclear. The present study demonstrates that the IDR positively and negatively regulates Clr4 function as follows: (i) the IDR interacts with the C-terminal catalytic domain and autoinhibits the methyltransferase activity of Clr4; (ii) the IDR cooperates with the RNAi pathway to spread H3K9me to adjacent regions; (iii) the IDR has RNA-binding ability that modulates the methyltransferase activity of Clr4; and (iv) the IDR has nucleosome-binding ability that facilitates targeting of chromatin substrates.

Our findings indicate that the interaction between the IDR and the catalytic domain autoinhibits the enzymatic activity of Clr4. Since the basic residues in the IDR play a critical role in this interaction (Fig. 2), it is likely that this interaction is mediated by electrostatic forces between charged residues. This idea is supported by our results that the interaction was weakened under high salt conditions (Supplementary Fig. S2C). Considering that the C-terminal catalytic domain contains the H3 tail-binding site formed by acidic residues [33], the basic residues in the IDR are likely to interact directly with acidic residues, thereby interfering with the interaction between the H3 tail and C-terminal catalytic domain. Although the C-terminal ARL has also been proposed to occlude the H3 tail-binding site [31], it is currently unclear how the N-terminal IDR and the C-terminal ARL work together to inhibit the H3 tail binding. The H3 tail-binding site may be sequentially occluded by the IDR and C-terminal ARL to achieve a multistep inhibition of Clr4 enzymatic activity.

Although the 3D structures of the CD and catalytic domain of Clr4 have been reported [47, 48], the structure of full-length Clr4, which verifies the interaction between the N-terminus and C-terminal catalytic domain, is not currently available. A recent study determined the structure of a complex of Clr4 and H3K9-methylated nucleosomes by cryo-electron microscopy [49], but the interaction between the N-terminus and C-terminal catalytic domain is not clearly observed in the solved structure. This suggests that the interaction is lost when Clr4 binds to H3K9-methylated nucleosomes. Further structural studies of complexes of full-length Clr4 and multi-nucleosomes with or without H3K9me may help elucidate the interaction between the N-terminus and C-terminal catalytic domain and the dynamic structural changes of Clr4. The Clr4 mutants used in this study may help understand the dynamic conformational changes of Clr4.

**Figure 6.**
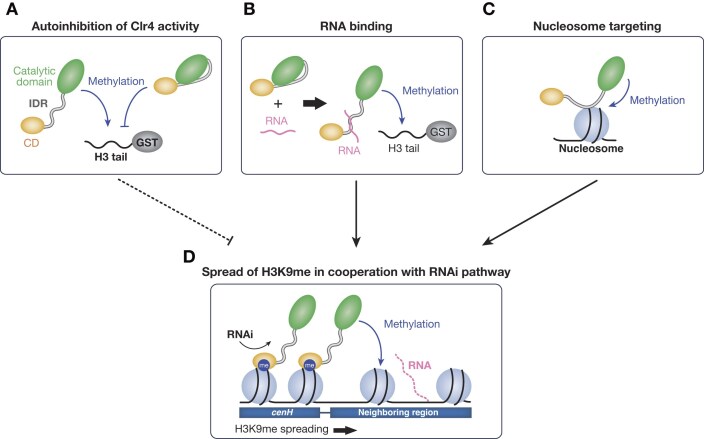
Model showing the Clr4 IDR functions in heterochromatin assembly. (**A**) The Clr4 IDR interacts with its C-terminal catalytic domain, which autoinhibits Clr4’s methyltransferase activity. (**B**) This autoinhibition is attenuated when the Clr4 IDR binds to RNA. (**C**) The IDR contributes to the targeting of nucleosomal substrates, allowing Clr4 to methylate H3K9 of nucleosomes. (**D**) At the *mat* locus, H3K9 methylation at the *cenH* nucleation center is introduced by the cooperative function of RNAi and Clr4. Once H3K9me is deposited, Clr4 activity increases due to its IDR binding to RNA and/or nucleosomes. This Clr4 activation facilitates the spreading of H3K9me from *cenH* to the surrounding regions. The multifunctionality of the Clr4 IDR contributes to heterochromatin spreading.

In this study, we showed that the basic residues of the IDR are critical for the spreading of H3K9me and that cells expressing mutant Clr4 with amino acid substitutions of the basic residues showed a silencing defect (Fig. 3). Since the amino acid substitutions in the IDR affect not only the interaction between the N-terminus and C-terminal catalytic domain, but also the RNA-binding and nucleosome-binding abilities of Clr4, the silencing defect observed in the mutant cells is likely the result of the combined effect of these multiple functions of the Clr4 IDR (Fig. 6). In our previous study demonstrating the importance of H3K14ub in promoting Clr4 methyltransferase activity, we showed that a portion of the IDR (residues 63–127) plays a critical role in discriminating ubiquitylated histone H3 and that cells expressing mutant Clr4 (Clr4Δ63–127) exhibited a silencing defect [32]. Since the deleted portion contains the basic residues mutagenized in this study, the silencing defect observed in the previous study may be caused not only by a deficiency in the recognition of ubiquitylated histone H3 but also by an impairment of the IDR function, including H3K9me spreading.

Herein, we also assessed how the Clr4 IDR facilitates H3K9me spreading. We found that Clr4 binds to RNA transcribed from the inserted marker gene (*mat3M::ade6*^+^) and that the basic residues in the IDR are required for the RNA binding (Fig. 4C). These results suggest that nascent RNAs transcribed from heterochromatic regions direct the interaction of Clr4 and RNAi pathway, and thereby Clr4 deposits and spreads H3K9me. The interaction between Clr4 and nascent RNAs may also be supported by additional machinery such as MTREC, which reportedly interacts with pericentromeric long noncoding RNA and recruits Clr4 to the pericentromeric region [50]. Previously, we showed that the Clr4 CD binds to RNAs in the presence of the H3K9me3 peptide and that basic residues in the C-terminal α-helix region of the CD are involved in the binding [38]. Here, we demonstrated that full-length Clr4 bound to RNA via the basic residues in the IDR without H3 peptides (Fig. 4). Presently, it is unclear how the RNA-binding activity of the CD contributes to the overall function of Clr4, but RNA-binding activities involving the CD and IDR may function cooperatively in the spreading of H3K9me. In addition to RNA binding, the IDR interacts with the C-terminal catalytic domain and autoinhibits the methyltransferase activity of Clr4 (Fig. 2). Considering that the appropriate molar ratio of RNA promotes the methyltransferase activity of Clr4 (Fig. 4E), it is conceivable that RNA interferes with the interaction between the IDR and C-terminal catalytic domain, facilitates the conformational change of Clr4, and alleviates the autoinhibition (Fig. 6B). Interestingly, we also found that a higher molar ratio of RNA inversely inhibited the methyltransferase activity of Clr4 (Fig. 4E). The excess amount of RNA may simply inhibit the binding between Clr4 and its substrates. However, this result may be related to the previous observation that accumulation of RNA on chromatin disrupts heterochromatin assembly and that RNA on chromatin must be degraded by the Ccr–Not complex for heterochromatin assembly [51].

In addition to RNA binding, the IDR contributes to the interaction between Clr4 and nucleosomes. This nucleosome-binding activity of Clr4 would also enhance H3K9me spreading *in vivo*. Notably, we found that although Clr4-C and Clr4 mutants with amino acid substitutions in the IDR exhibited higher methyltransferase activity than full-length Clr4 when H3N-GST was used as a substrate (Fig. 2E), they showed significantly reduced activity when nucleosomes were used as a substrate (Fig. 5C). The reason for this effect could be that the IDR simply stabilizes the interaction between Clr4 and nucleosomes. Alternatively, given that H3 tails are thought to be associated with nucleosomal DNA, thereby preventing the interaction between the catalytic domain of Clr4 and H3 tails, it is possible that the Clr4 IDR facilitates the release of the H3 tail from nucleosomal DNA, thereby allowing the catalytic domain of Clr4 to access H3K9. Notably, H3K9me2 was partially retained in *clr4* mutant strains (Fig. 3 and Supplementary Figs S6, S8, and S9), suggesting that mutant Clr4 does not completely lose the methyltransferase activity for nucleosomes *in vivo* and that other mechanisms would compensate for the deficiency.

Besides RNA, there may be several other mechanisms that can alter the conformation to alleviate Clr4 autoinhibition. Modifications of Clr4 itself are considered as candidates. For example, in this study, the basic residues in the IDR were replaced by glutamine to investigate their importance in the IDR function. Because glutamine mimics an acetylated lysine, acetylation of lysine residues in the IDR could disrupt the interaction, although such modifications have not been described previously. In addition to acetylation, SUMOylation [52, 53] or ubiquitination [54] could also act as a trigger of conformational changes of Clr4. These modifications could be regulated in a cell cycle-dependent manner, as a recent study showed that meiosis-specific phosphorylation by Cdk1 at S458 in the C-terminal ARL counteracts the transition from H3K9me2 to H3K9me3 [55]. Another potential candidate for a conformational switch is liquid–liquid phase separation (LLPS). A number of proteins with extended IDRs has been reported to be able to form condensates. LLPS has been implicated in heterochromatin assembly, as the LLPS activity of the HP1 protein is required for chromatin condensation [56, 57]. A recent study showed that Clr4 is able to form condensates with Swi6 (HP1 homologue in *S. pombe*) and that the formation of liquid droplets promotes the methyltransferase activity of Clr4 [54]. The conditions of the condensate could alter the structure and autoinhibitory state of Clr4. Other proteins or their modifications may also be involved in the regulation of Clr4 activity. We previously showed that Clr4 activity is promoted by histone H3 ubiquitination [32]. In addition, Clr4 reportedly interacts with variable proteins, such as CLRC components [58–62], Swi6 [63], and ATF/CREB [27]. It is possible that these proteins act as triggers to switch the conformation of Clr4.

## Supplementary Material

gkaf878_Supplemental_Files

## Data Availability

The authors declare that the data supporting the findings of this study are available within the paper and its supplementary information file.
