# Supplementary material for: Intrinsically disordered region of Clr4/Suv39 regulates its enzymatic activity and ensures heterochromatin spreading
Source: Nucleic Acids Res. 2025 Sep 9;53(17):gkaf878. doi: 10.1093/nar/gkaf878 (PMC12418378; doi:10.1093/nar/gkaf878)
Supplement: gkaf878_Supplemental_Files [file gkaf878_supplemental_files.zip › Supplementary Figures_R1.pdf]

Supplementary figures

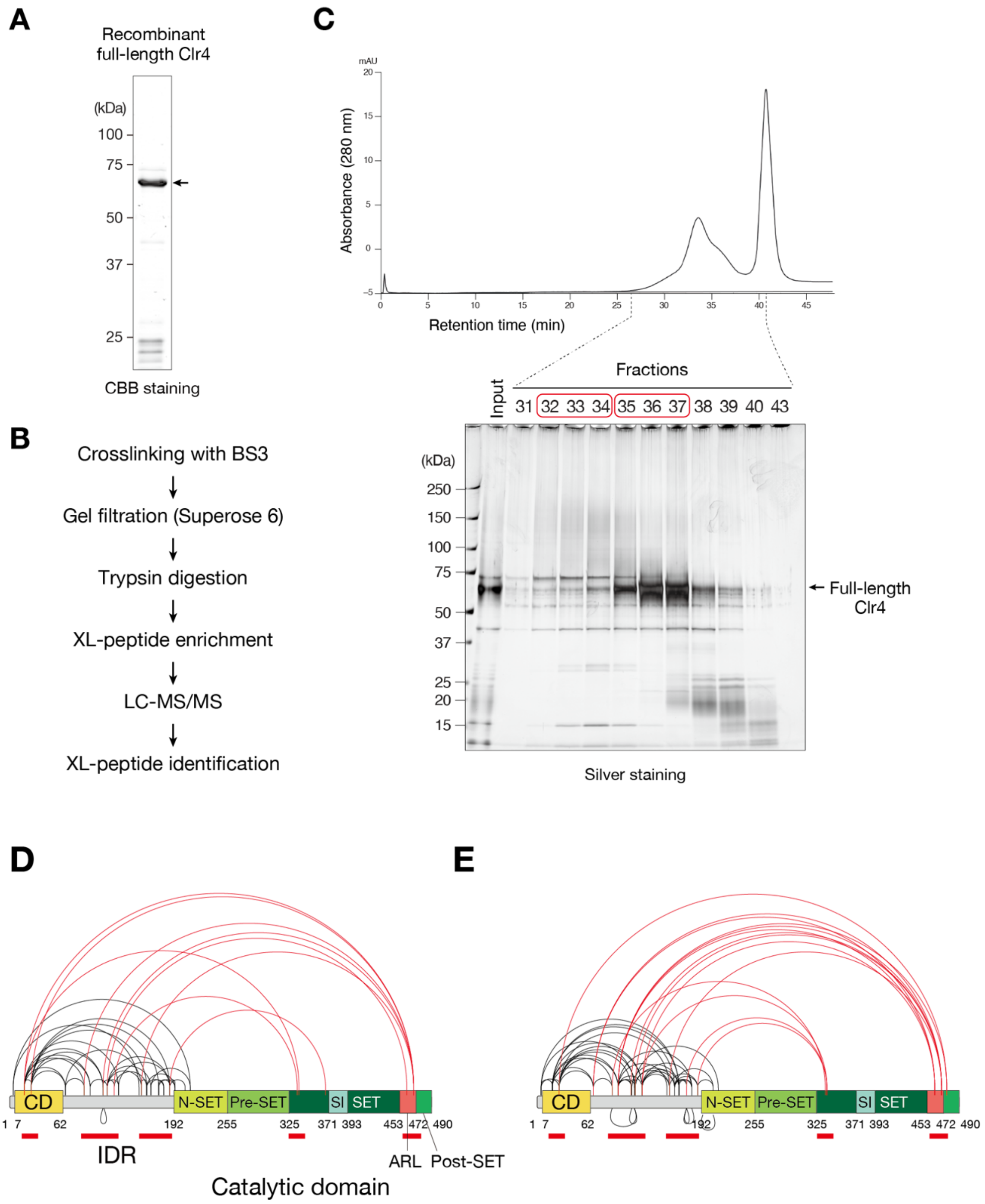

Supplementary Figure S1. Interactions between the N- and C-terminal regions of Clr4 detected by cross-linking mass spectrometry (XL-MS)

(**A**) Recombinant protein used in XL-MS was resolved by 10% SDS-PAGE and visualized by CBB staining. (**B**) Outline of the XL-MS. (**C**) Chromatogram and elution profile of the cross-linked Clr4 by gel filtration chromatography. An elution profile for Clr4 is shown (top), and the eluted proteins were resolved by SDS-PAGE using 4–20% gradient gel and visualized by silver staining (bottom). Fractions used in XL-MS analysis are indicated by red boxes. (**D and E**) Representative result of crosslinking-MS using full-length Clr4 prepared from early (Fractions 32–34, D) and late (Fractions 35–37, E) eluates. Black and red arcs indicate visualized crosslinks within the N-terminal region and between the N- and C-terminal regions, respectively. Each edge of arcs indicates a XL residue. The major regions containing lysine residues cross-linked between the N-terminal and C-terminal regions of Clr4 are indicated below by red lines.

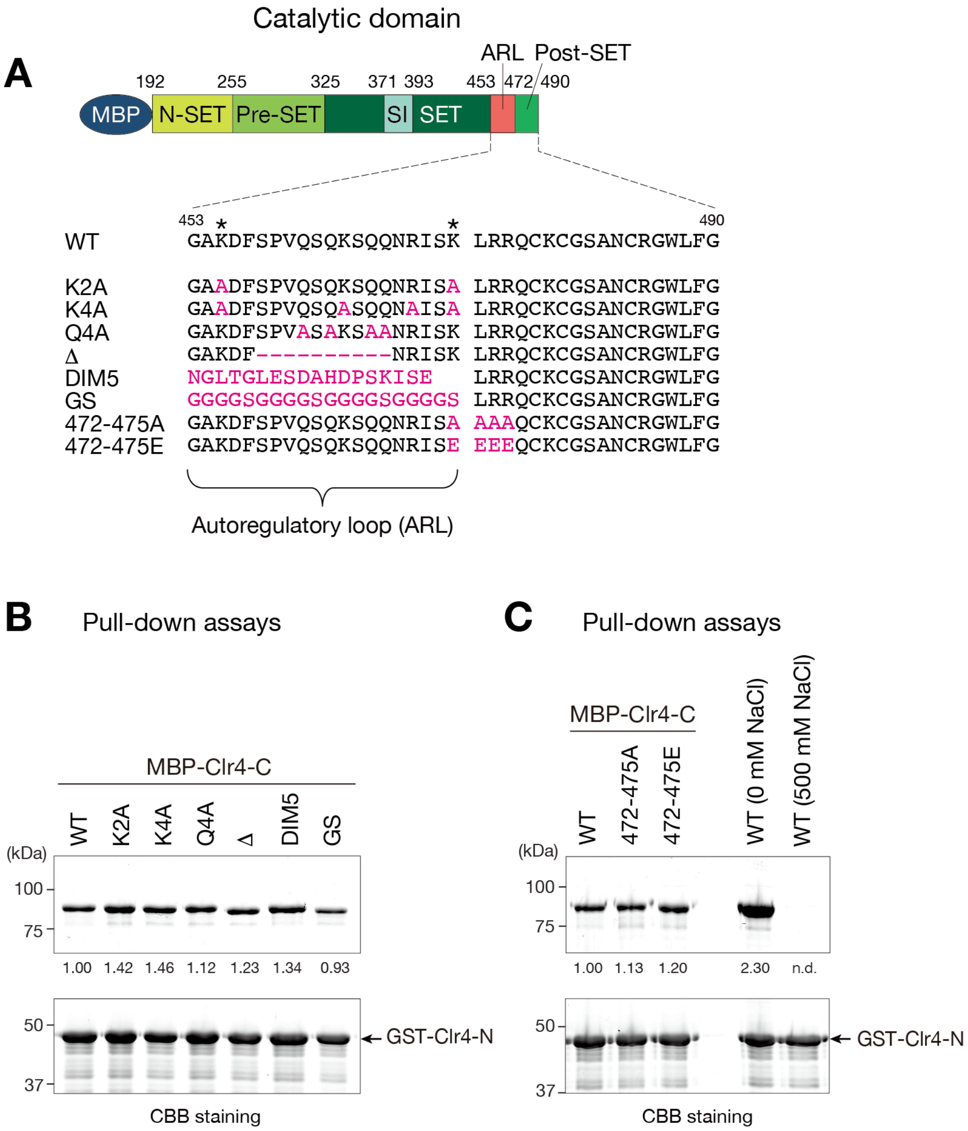

Supplementary Figure S2. Amino acid residues within or in close proximity to the C-terminal autoregulatory loop are not essential for the interaction with the N-terminal region of Clr4

(**A**) A schematic diagram and sequence alignment of the recombinant Clr4. The amino acid residues within or in close proximity to the C-terminal autoregulatory loop (ARL) were mutated as indicated. DIM5; Clr4 homologue in *Neurospora crassa*. Lysine residues subjected to automethylation are indicated by asterisks. (**B and C**) Results of GST pull-down assays using the recombinant Clr4 proteins shown in (A). The signals of pulled-down MBP-Clr4-C were calculated and relative fold enrichments are shown beneath each lane.

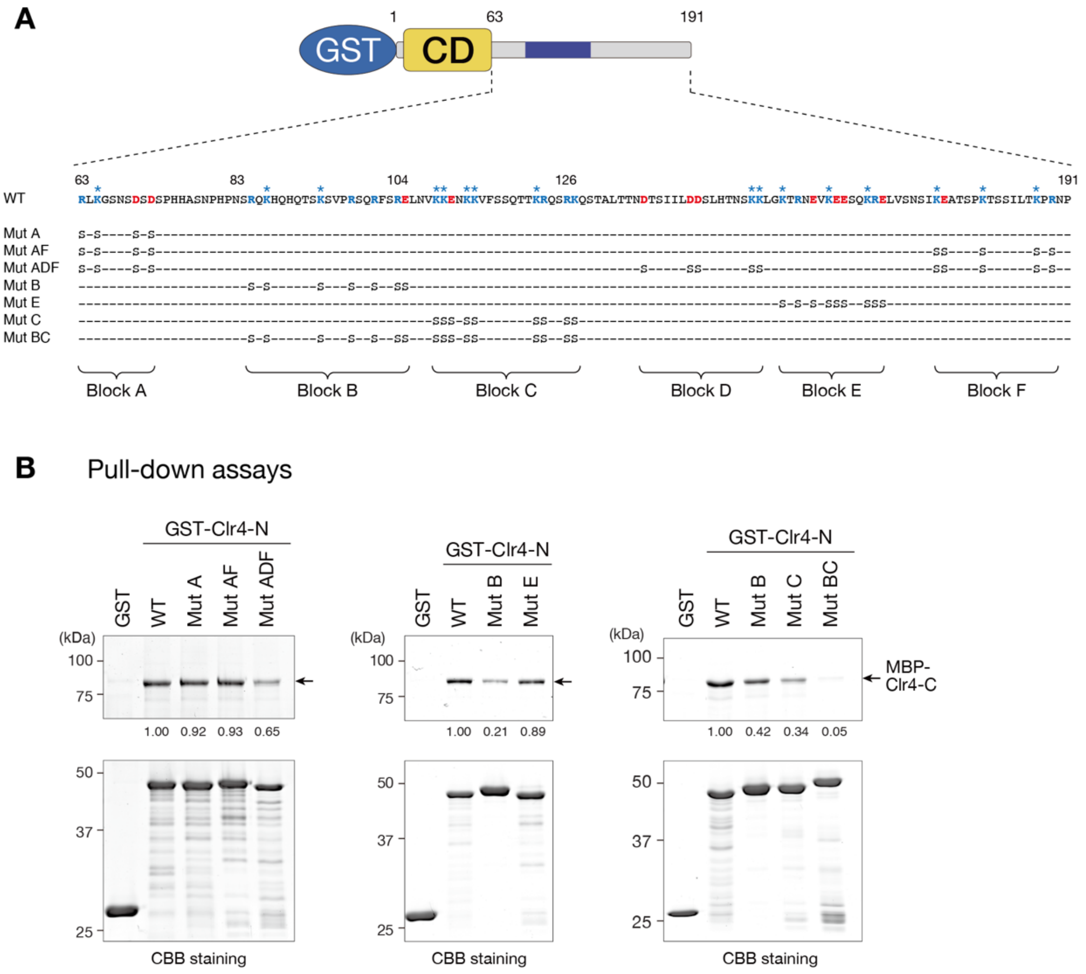

Supplementary Figure S3. Basic residues in the Clr4 IDR are involved in the interaction with the C-terminal catalytic domain

(**A**) A schematic diagram and alignment of the N-terminal recombinant Clr4. The IDR was subdivided into six blocks (designated as blocks A-F), and the charged residues were replaced with serine. Basic and acidic residues are indicated in blue and red, respectively. Blue box in the diagram shows block B and C. Lysine residues cross-linked to the C-terminal region in the XL-mass (Supplementary Table S1 and S2) are indicated by asterisks. (**B**) Results of GST pull-down assays using recombinant Clr4 proteins used in (A), showing that the substitution of charged residues in block B or C largely affected the interaction with the C-terminal region.

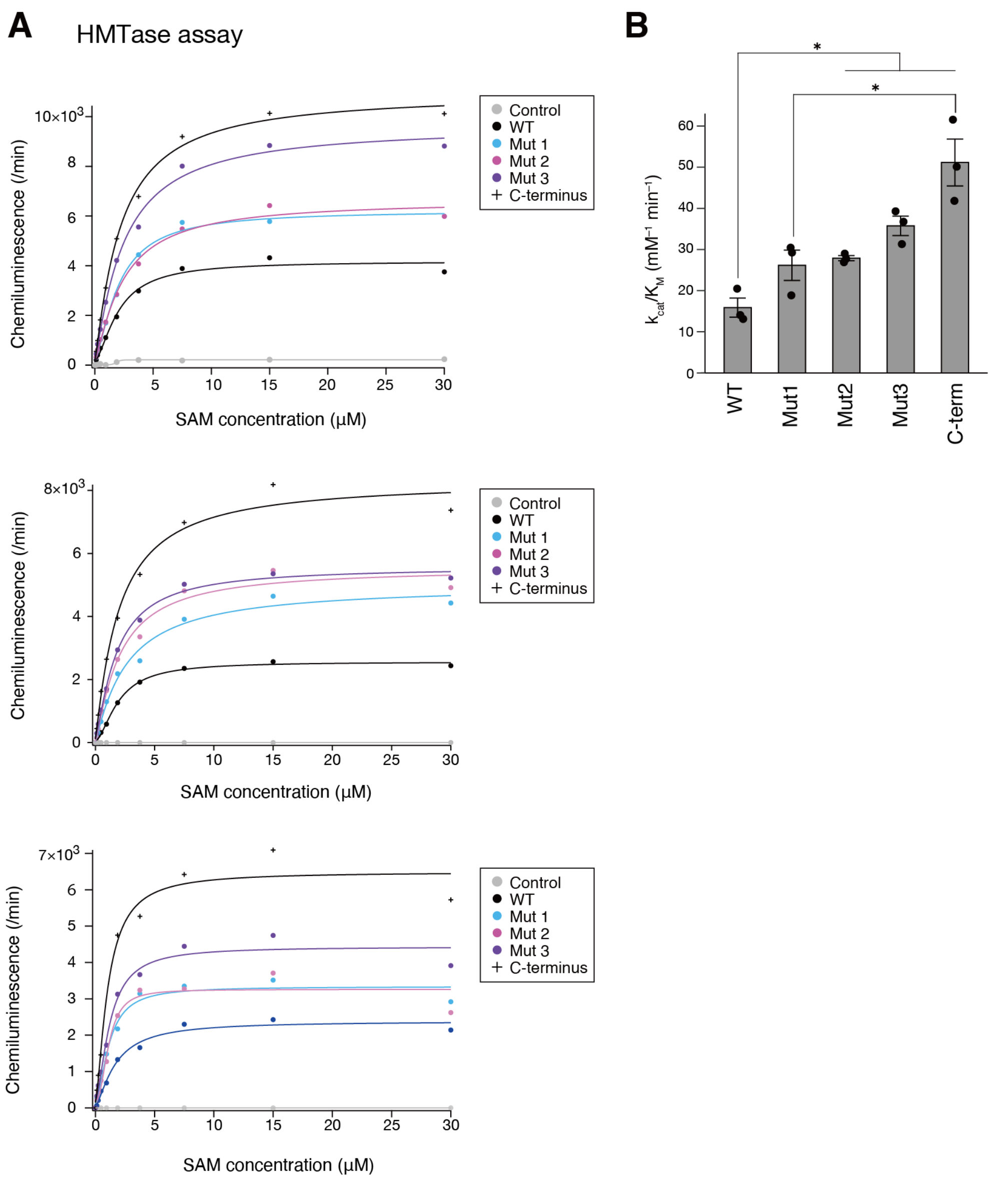

Supplementary Figure S4. Basic residues in the Clr4 IDR are involved in the autoinhibition of its enzymatic activity

(**A**) Results of in vitro MTase assays using wild-type, mutant or N-terminally deleted Clr4. H3 peptides were used as substrates, and the MTase-Glo Methyltransferase Assay Kit (Promega) was used to evaluate Clr4 activity. Triplicate data are shown. (**B**) k_{cat}/K_M (mM^{-1} min^{-1}) values were calculated. Statistical significance was determined using a two-tailed unpaired Student's t-test. *$p<0.05$, **$p<0.005$, ***$p<0.0005$. Error bars: SD; n=3.

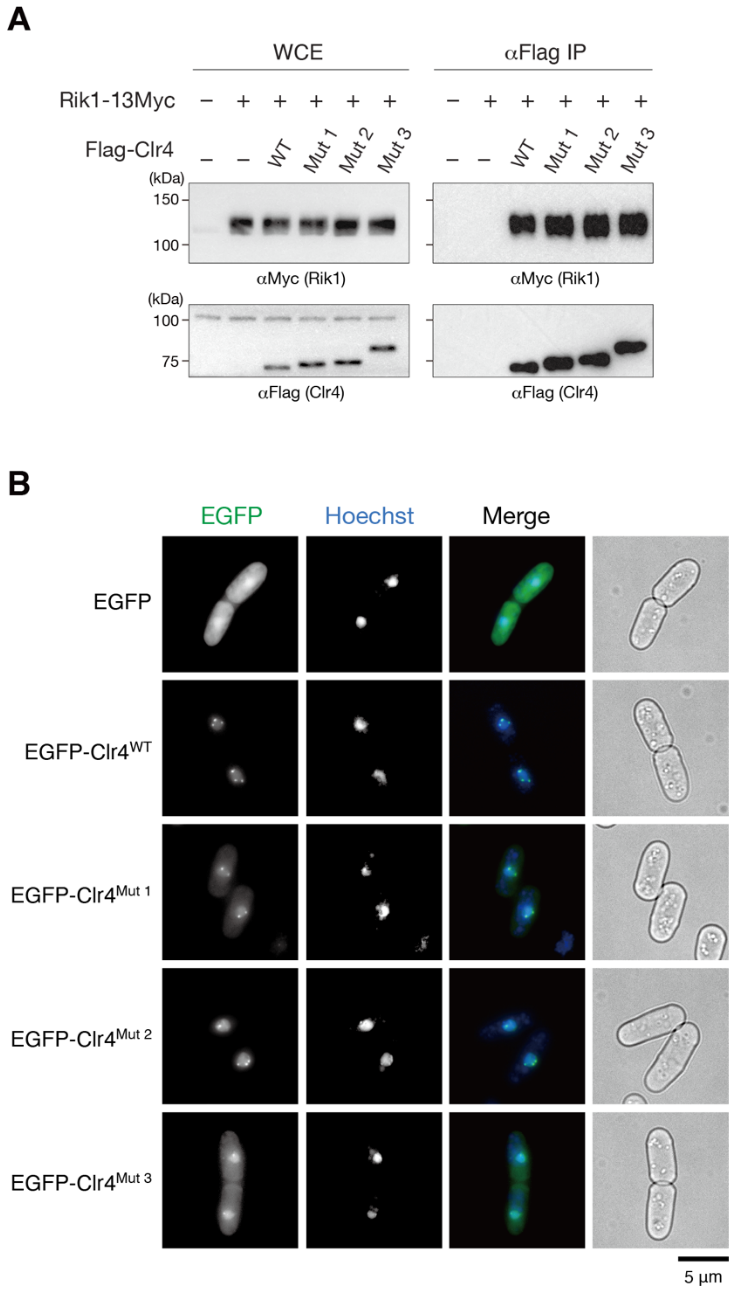

Supplementary Figure S5. Effects of the mutant Clr4 on CLRC formation and its nuclear localization

(**A**) Whole cell lysates (input) of control cells and cells expressing FLAG-tagged wild type or mutant Clr4 were subjected to immunoblotting using the indicated antibodies. The anti-FLAG immunoprecipitates (IP) were also analyzed. (**B**) Representative images showing the nuclear localization of EGFP-fused Clr4.

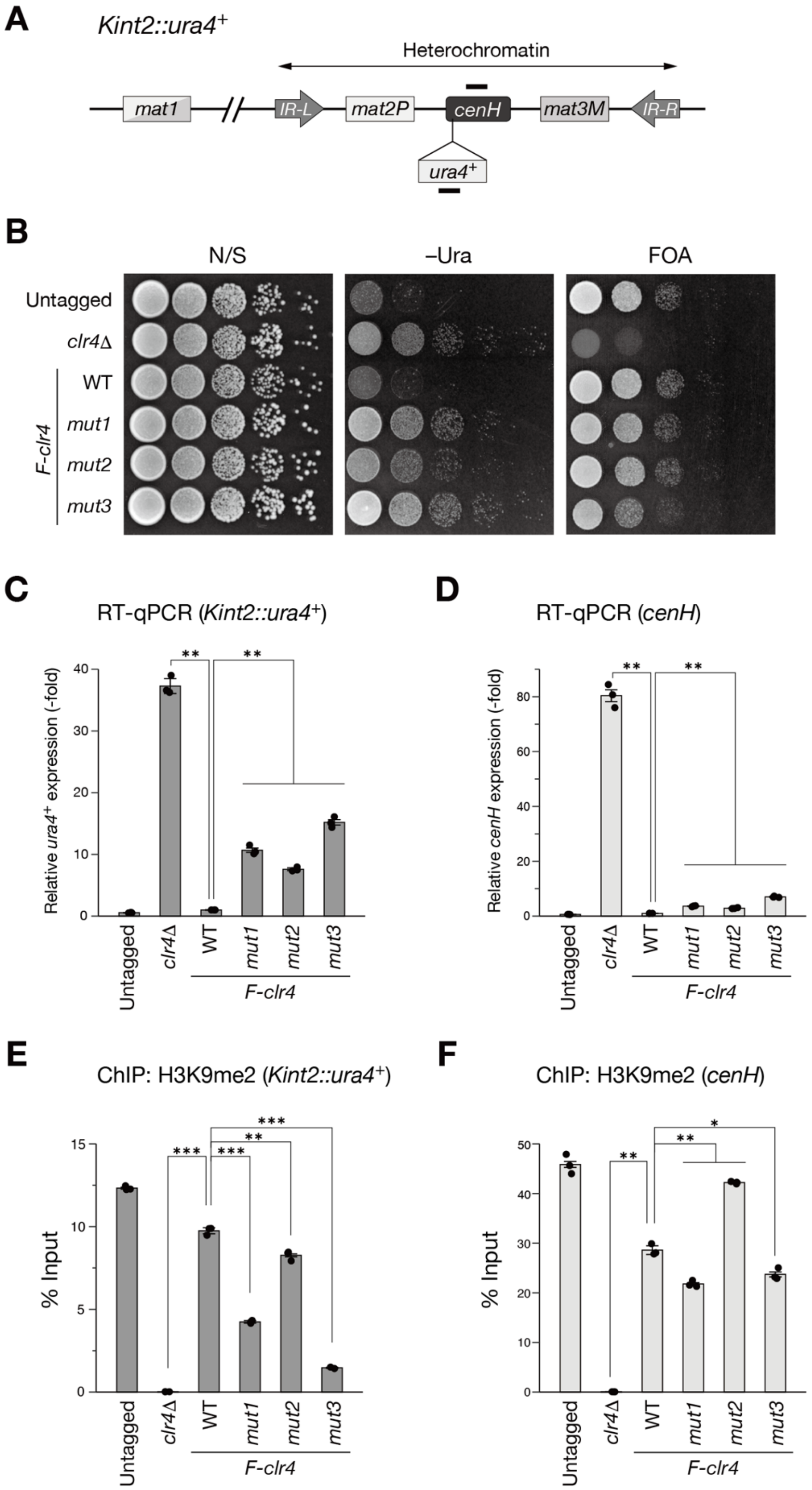
A
Kint2::ura4+
Heterochromatin
mat1
IR-L
mat2P
cenH
mat3M
IR-R
ura4+
B
N/S
–Ura
FOA
Untagged
clr4Δ
WT
mut1
mut2
mut3
F-clr4
C
RT-qPCR (Kint2::ura4+)
Relative ura4+ expression (-fold)
D
RT-qPCR (cenH)
Relative cenH expression (-fold)
E
ChIP: H3K9me2 (Kint2::ura4+)
% Input
F
ChIP: H3K9me2 (cenH)
% Input

Supplementary Figure S6. Effects of mutant Clr4 on heterochromatic gene silencing at the mating-type Kint2 locus

(**A**) A schematic diagram of the mating-type locus showing the *Kint2::ura4*$^{+}$ insertion site. Black bars indicate the target regions of ChIP-qPCR and RT-qPCR for *Kint2::ura4*$^{+}$ and *cenH*. (**B**) Heterochromatic silencing assays of control cells (*clr4*$^{+}$), *clr4*Δ cells, and cells expressing FLAG-tagged wild-type (*F-clr4*^{WT}) or mutant Clr4 (*F-clr4*^{mut1}, *F-clr4*^{mut2}, *F-clr4*^{mut3}). Silencing at *Kint2::ura4*$^{+}$ was evaluated. Ten-fold serial dilutions of the indicated strains were spotted onto non-selective medium (N/S), medium lacking uracil (–Ura) and medium containing 5-FOA (FOA). (**C and D**) Levels of *Kint2::ura4*$^{+}$ (C) and *cenH* (D) transcripts were quantified by RT-qPCR, relative to the control cell (*F-clr4*^{WT}). (**E and F**) ChIP analysis of H3K9me2 levels at *Kint::ura4*$^{+}$ (E) and *cenH* locus (F), relative to the control *act1*$^{+}$. In (C)–(F), statistical significance was determined using a two-tailed unpaired Student's t-test. *$p<0.05$, **$p<0.005$, ***$p<0.0005$. Error bars: SD; n=3.

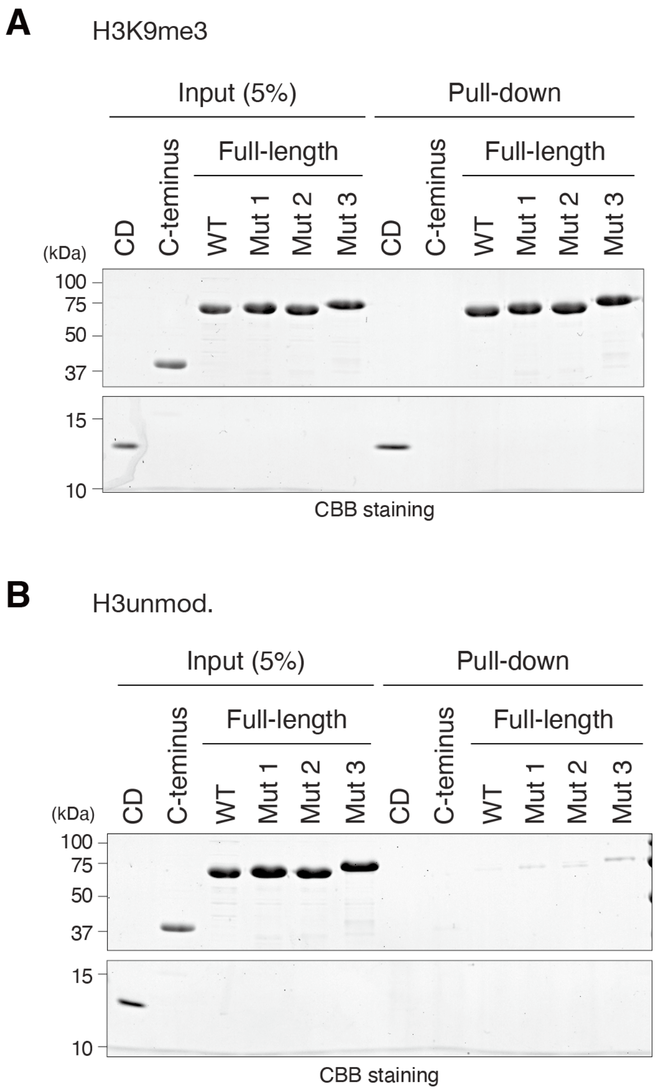

Supplementary Figure S7. The mutant Clr4 proteins bind to H3K9me with an affinity comparable to that of the wild-type Clr4 protein.

(**A and B**) Representative results of peptide pull-down assays using wild-type (WT), mutant, or N-terminally deleted Clr4. Clr4 CD was used as a control. Proteins pulled down with the K9-trimethylated H3 (H3K9me3) peptide (H3K9me3) (A) or the unmodified H3 peptide (H3unmod.) (B) were resolved by SDS-PAGE and visualized by Coomassie staining.

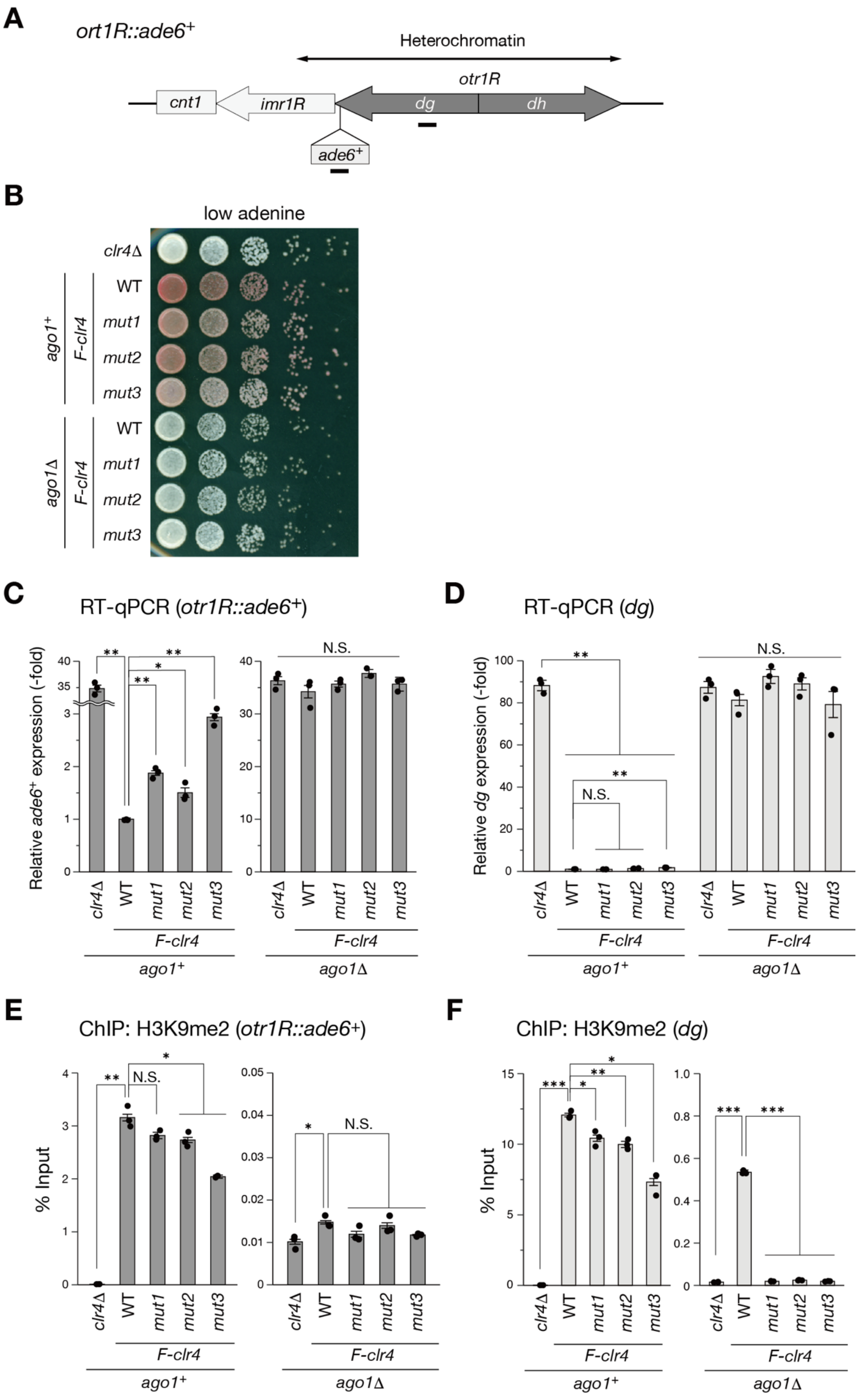
A
ort1R::ade6+
Heterochromatin
otr1R
cnt1
imr1R
dg
dh
ade6+
B
low adenine
clr4Δ
WT
mut1
mut2
mut3
ago1+
F-clr4
ago1Δ
C
RT-qPCR (otr1R::ade6+)
Relative ade6+ expression (-fold)
N.S.
D
RT-qPCR (dg)
Relative dg expression (-fold)
E
ChIP: H3K9me2 (otr1R::ade6+)
% Input
F
ChIP: H3K9me2 (dg)

Supplementary Figure S8. Effects of mutant Clr4 on heterochromatic gene silencing at the pericentromeric region

(**A**) A schematic diagram of the centromeric region of chromosome 1 and *otr1R::ade6⁺* insertion site. Black bars indicate the target regions of ChIP-qPCR and RT-qPCR for *otr1R::ade6⁺* and *dg*. (**B**) Heterochromatic silencing assays of *clr4Δ* cells and cells expressing FLAG-tagged wild-type ($F\text{-}clr4^{WT}$) or mutant Clr4 ($F\text{-}clr4^{mut1}$, $F\text{-}clr4^{mut2}$, $F\text{-}clr4^{mut3}$). Silencing at the $otr1R::ade6^{+}$ was evaluated. Ten-fold serial dilutions of the indicated strains were spotted onto adenine-depleted medium. (**C and D**) Levels of $otr1R::ade6^{+}$ (C) and *dg* (D) transcripts were quantified by RT-qPCR, relative to the control cell ($F\text{-}clr4^{WT}$). (**E and F**) ChIP analysis of H3K9me2 levels at the $otr1R::ade6^{+}$ (E) and the *dg* locus (F), relative to the control $act1^{+}$. In (C)–(F), statistical significance was determined using a two-tailed unpaired Student's t-test. Not significant (NS), p>0.05, *p<0.05, **p<0.005, ***p<0.0005. Error bars: SD; n=3.

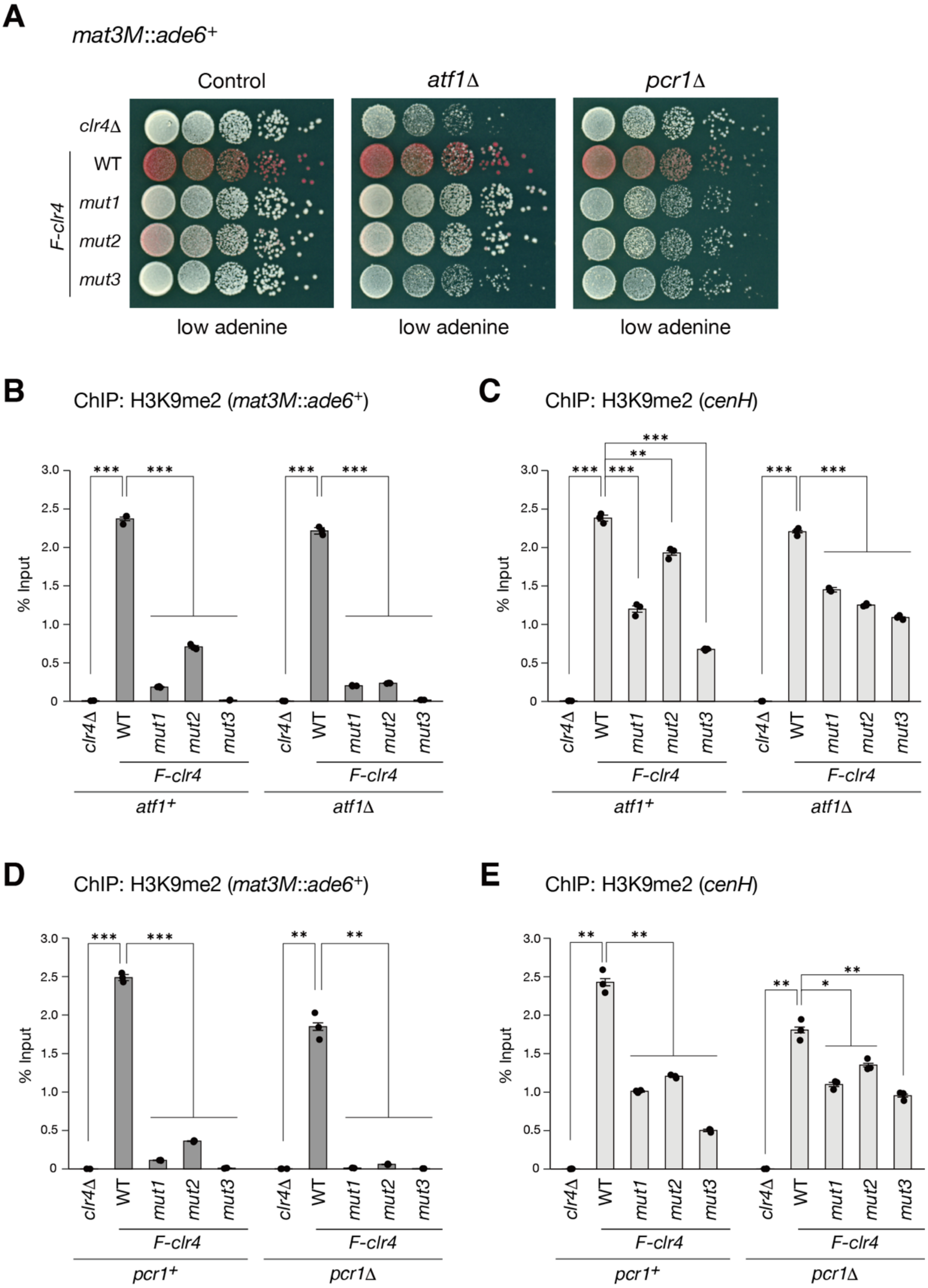
A
mat3M::ade6+
Control
atf1Δ
pcr1Δ
clr4Δ
WT
mut1
mut2
mut3
F-clr4
low adenine
low adenine
low adenine
B
ChIP: H3K9me2 (mat3M::ade6+)
C
ChIP: H3K9me2 (cenH)
D
ChIP: H3K9me2 (mat3M::ade6+)
E
ChIP: H3K9me2 (cenH)
% Input
0
0.5
1.0
1.5
2.0
2.5
3.0
clr4Δ
WT
mut1
mut2
mut3
F-clr4
atf1+
atf1Δ
pcr1+
pcr1Δ
***
**
*

Supplementary Figure S9. Neither Atf1 nor Pcr1 plays a cooperative role with the Clr4 IDR in the heterochromatin nucleation at the *cenH* locus

(**A**) Heterochromatic silencing assays of cells lacking *clr4* (*clr4*Δ), and cells expressing FLAG-tagged wild-type ($F\text{-}clr4^{WT}$) or mutant Clr4 ($F\text{-}clr4^{mut1}$, $F\text{-}clr4^{mut2}$, $F\text{-}clr4^{mut3}$) in combination with *atf*1 or *pcr1* deletion (*atf1*Δ or *pcr1*Δ). Silencing at $mat3M::ade6^{+}$ was evaluated. Ten-fold serial dilutions of the indicated strains were spotted onto adenine-limited medium. (**B and C**) ChIP analysis of H3K9me2 levels at $mat3M::ade6^{+}$ (B) and the *cenH* locus (C) in control and *atf1*Δ strains. (**D and E**). ChIP analysis of H3K9me2 levels at $mat3M::ade6^{+}$ (D) and *cenH* locus (E) in control and *pcr1*Δ strains. In (B)–(E), statistical significance was determined using a two-tailed unpaired Student's t-test. *$p<0.05$, **$p<0.005$, ***$p<0.0005$. Error bars: SD; n=3.

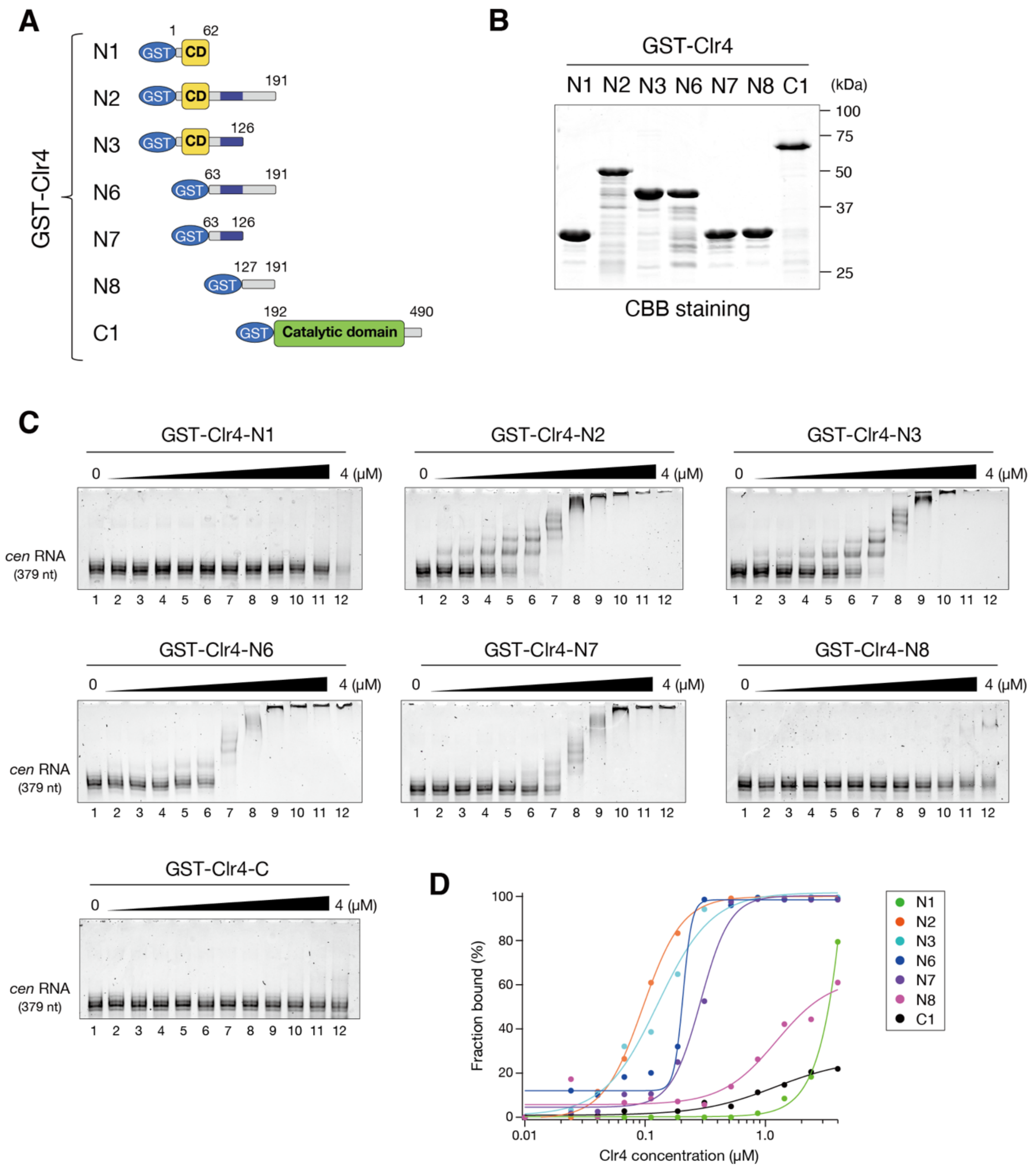

Supplementary Figure S10. The region within the Clr4 IDR containing blocks B and C is required for RNA binding

(**A**) Schematic diagram of GST-fused Clr4. Blue boxes indicate the Clr4 IDR region, which contains blocks B and C. **(B)** Recombinant Clr4 proteins used in (C) were resolved by SDS-PAGE and visualized by CBB staining. (**C**) Representative EMSA results using GST-fused Clr4 shown in (A) and (B). A 379 nt single-stranded RNA, corresponding to pericentromeric repeat, was used as a probe. (**D**) Quantification was performed by calculating the fraction of retarded RNA probes, which was then plotted against each protein concentration.

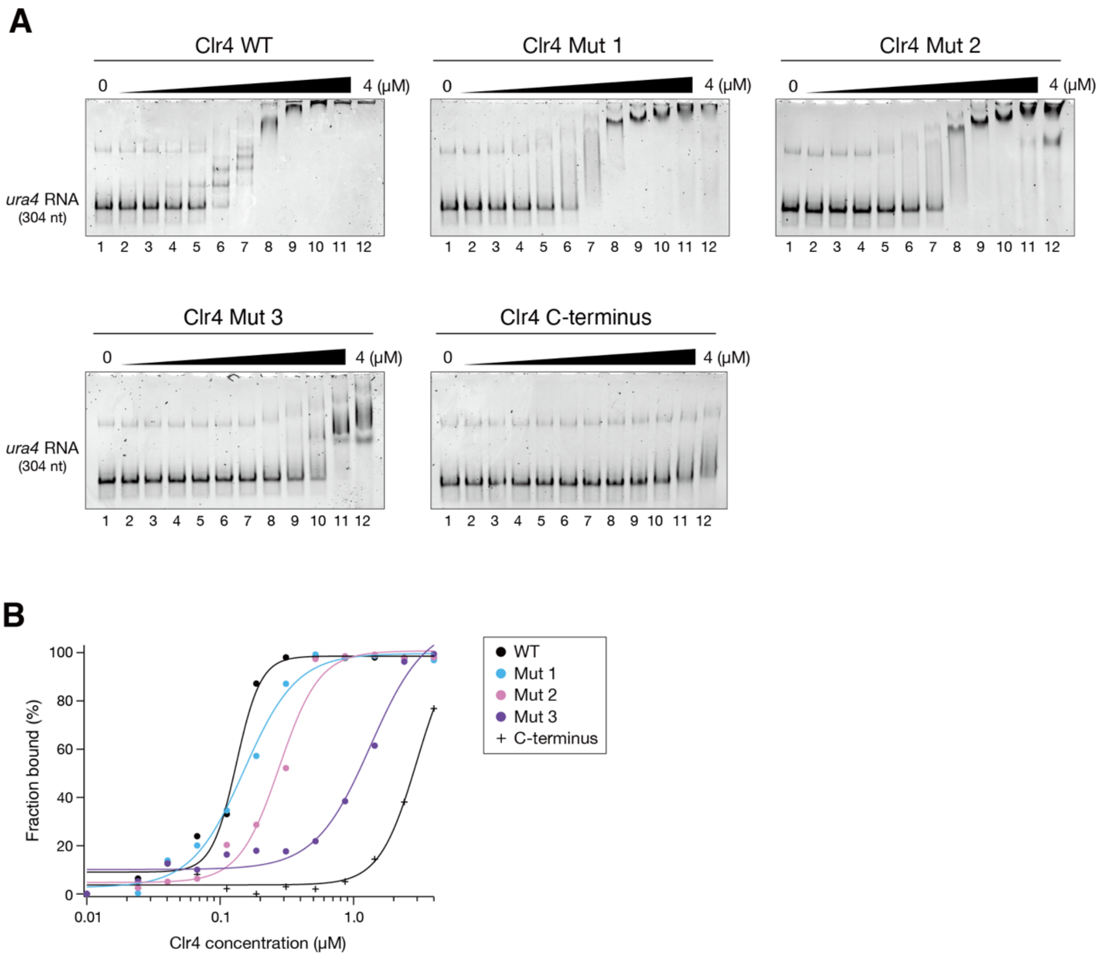

Supplementary Figure S11. Clr4 binds to RNA without particular sequence specificity

(**A**) Representative EMSA results using wild-type, mutant or N-terminally deleted Clr4. A 304 nt single-stranded RNA, corresponding to *ura4*$^{+}$ gene, was used as a probe. (**B**) Quantification was performed by calculating the fraction of retarded RNA probes, which was then plotted against each protein concentration.

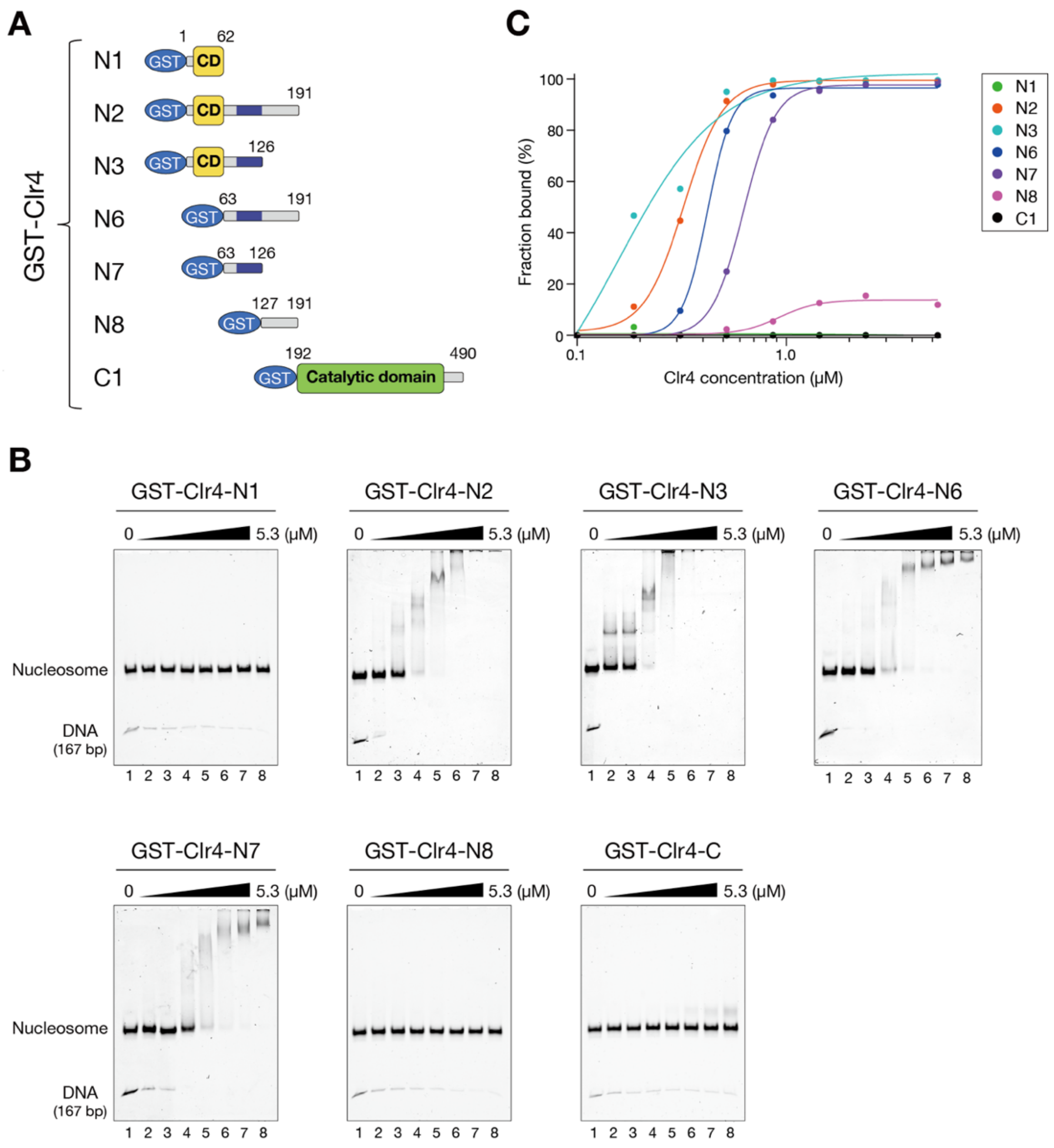

Supplementary Figure S12. The region within the Clr4 IDR containing blocks B and C is required for nucleosome binding

(**A**) Schematic diagram of GST-fused Clr4. (**B**) Representative EMSA results using the GST-fused Clr4. Reconstituted mono-nucleosomes containing 167 bp DNA were used as the probe. (**C**) Quantification was performed by calculating the fraction of retarded mono-nucleosomes, which was then plotted against each protein concentration.

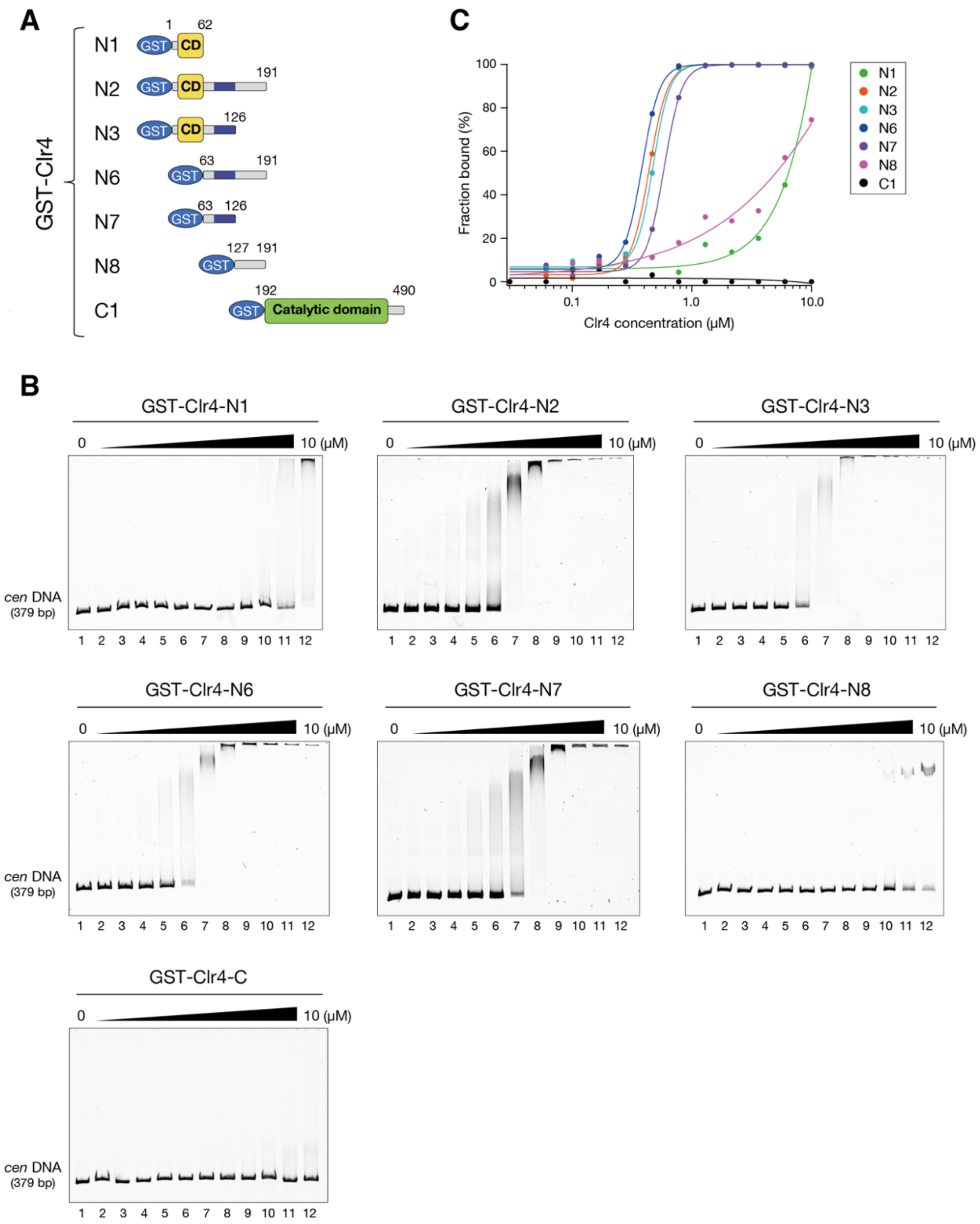

Supplementary Figure S13. The region within the Clr4 IDR containing blocks B and C is required for DNA binding

(**A**) Schematic diagram of GST-fused Clr4. (**B**) Representative EMSA results using the GST-fused Clr4. A 379 bp double-stranded DNA corresponding to the pericentromeric repeat was used as the probe. (**C**) Quantification was performed by calculating the fraction of retarded DNA probe, which was then plotted against each protein concentration.

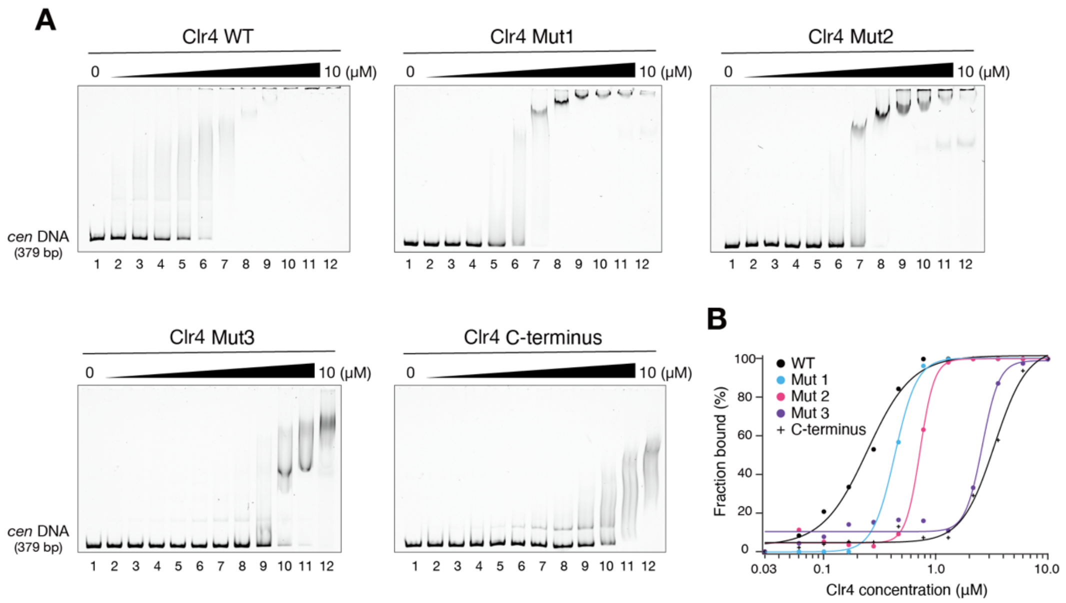

Supplementary Figure S14. Basic residues of the Clr4 IDR are essential for DNA binding

(**A**) Representative results of EMSA using wild-type, mutant or N-terminally deleted Clr4. A 379 bp double-stranded DNA corresponding to the centromeric repeat, was used as a probe. **(B)** Quantification of EMSA results in (A); the fraction of retarded DNA probe was plotted against each protein concentration.
