# Supplementary material for: Intrinsically disordered region of Clr4/Suv39 regulates its enzymatic activity and ensures heterochromatin spreading
Source: Nucleic Acids Res. 2025 Sep 9;53(17):gkaf878. doi: 10.1093/nar/gkaf878 (PMC12418378; doi:10.1093/nar/gkaf878)
Supplement: gkaf878_Supplemental_Files [file gkaf878_supplemental_files.zip › Supplementary Tables_R1.pdf]

Supplementary Table S1. List of cross-linked peptides identified in Fractions 32–34

Id	Score	Peptide 1		Peptide 2		Intra- or Inter molecular
		Sequence	XL position	Sequence	XL position	
1	72.56	LKGSNSDSDSPHHASNPHPNSR	65	QKHQHQTSK	87	Intra or Inter
2	54.47	QKHQHQTSK	87	ISKLR	472	Intra or Inter
3	77.45	NEVKEESQKR	160	LGKTR	154	Intra or Inter
4	56.51	QKHQHQTSK	87	IVDEKLDR	17	Intra or Inter
5	54.44	KVFSSQTTK	114	EESQKR	165	Intra or Inter
6	57.84	GGSMSPKQEEYEVER	4	ELFRKKLR	211	Intra or Inter
7	74.6	LKGSNSDSDSPHHASNPHPNSR	65	IVDEKLDR	17	Intra or Inter
8	64.28	LKGSNSDSDSPHHASNPHPNSR	65	NGAVKLYR	25	Intra or Inter
9	58.07	HQHQTSKSVPR	94	KVFSSQTTK	114	Intra or Inter
10	68.4	NEVKEESQK	160	VFSSQTTKR	122	Intra or Inter
11	60.96	LKGSNSDSDSPHHASNPHPNSR	65	LGKTRNEVK	154	Intra or Inter
12	109.31	QKHQHQTSKSVPR	94	IVDEKLDR	17	Intra or Inter
13	98.81	HQHQTSKSVPR	94	NGAVKLYR	25	Intra or Inter
14	52.86	KVFSSQTTK	114	LGKTR	154	Intra or Inter
15	52.68	NPSKLDSYTHLSFYEK	193	NEVKEESQK	160	Intra or Inter
16	93.04	ISKLR	472	LGKTR	154	Intra or Inter
17	62.32	EATSPKTSSILTKPR	180	NEVKEESQKR	165	Intra or Inter
18	64.26	EATSPKTSSILTKPR	180	TRNEVKEESQK	160	Intra or Inter
19	52.59	VFSSQTTKR	122	ELNVKK	109	Intra or Inter
20	63.42	ELNVKK	109	ISKLR	472	Intra or Inter
21	55.95	SQRFSRELNVKKENKK	109	NGAVKLYRIR	25	Intra or Inter
22	60.61	NGAVKLYR	25	ISKLR	472	Intra or Inter
23	59.85	DFSPVQSQKSQQNR	464	KVFSSQTTK	114	Intra or Inter
24	56.31	IVDEKLDR	17	ELNVKK	109	Intra or Inter
25	55.85	IVDEKLDR	17	ISKLR	472	Intra or Inter
26	75.94	GRTLPLEIFKTKEKGWGVRSLR	338	IVDEKLDRNGAVKLYR	17	Intra or Inter
27	74.72	TSSILTKPR	187	NGAVKLYR	25	Intra or Inter
28	74.34	ELVSNSIKEATSPK	174	TSSILTKPR	187	Intra or Inter
29	82.26	KQSTALTTNDTSIILDDSLHTNSK	127	KVFSSQTTK	114	Intra or Inter
30	50.83	KLGKTRNEVKEESQKR	151	GGSMSPKQEEYEVER	4	Intra or Inter
31	56.23	ELVSNSIKEATSPKTSSILTKPR	187	KVFSSQTTKR	114	Intra or Inter
32	79.03	NPSKLDSYTHLSFYEKRELFR	205	TSSILTKPR	187	Intra or Inter
33	51.69	KQSTALTTNDTSIILDDSLHTNSK	127	IVDEKLDR	17	Intra or Inter
34	87.58	KQSTALTTNDTSIILDDSLHTNSK	127	TSSILTKPR	187	Intra or Inter
35	87.58	NPSKLDSYTHLSFYEK	193	TSSILTKPR	187	Intra or Inter
36	67.75	NPSKLDSYTHLSFYEK	193	ELVSNSIKEATSPK	174	Intra or Inter
37	57.84	RELVSNSIKEATSPK	174	LDRNGAVKLYRIR	25	Intra or Inter
38	52.3	TRNEVKEESQKRELVSNSIK	160	LDRNGAVKLYR	25	Intra or Inter
39	59.97	FSRELNVKKENKKVFSSQTTKR	122	TKEKGWGVRSLR	336	Intra or Inter
40	56.91	SVPRSQRFSRELNVKKENKKVFSSQTTKR	109	SQRFSRELNVKKENKKVFSSQTTKR	110	Inter
41	59.85	FAPAGTFITCYLGEVITSAEAAKR	369	TSSILTKPR	187	Intra or Inter
42	56.86	NPSKLDSYTHLSFYEKRELFRKK	211	KLGKTRNEVKEESQKR	165	Intra or Inter
43	62.34	IVDEKLDR	17	NGAVKLYR	25	Intra or Inter

Supplementary Table S2. List of cross-linked peptides identified in Fractions 35–37

Id	Score	Peptide 1		Peptide 2		Intra- or Inter molecular
		Sequence	XL position	Sequence	XL position	
1	58.77	HQHQTSKSVPRSQRFSR	94	RQCKCGSANCR	478	Intra or Inter
2	70.83	LKGSNSDSDSPHHASNPHPNSR	65	QKHQHQTSK	87	Intra or Inter
3	69.86	HQHQTSKSVPR	94	KENKK	110	Intra or Inter
4	52.96	KENKK	113	LGKTR	154	Intra or Inter
5	62	QKHQHQTSK	87	IVDEKLDR	17	Intra or Inter
6	57.84	GGSMSPKQEEYEVER	4	NGAVKLYRIR	25	Intra or Inter
7	59.04	LKGSNSDSDSPHHASNPHPNSR	65	ISKLR	472	Intra or Inter
8	52.68	HQHQTSKSVPR	94	VFSSQTTKR	122	Intra or Inter
9	61.26	GGSMSPKQEEYEVERIVDEK	4	EESQKR	165	Intra or Inter
10	103.41	RLKGSNSDSDSPHHASNPHPNSR	65	NGAVKLYR	25	Intra or Inter
11	54.77	HQHQTSKSVPR	94	KVFSSQTTK	114	Intra or Inter
12	62.53	HQHQTSKSVPR	94	ISKLR	472	Intra or Inter
13	53.36	HQHQTSKSVPR	94	ELNVKK	109	Intra or Inter
14	56.57	LKGSNSDSDSPHHASNPHPNSR	65	IVDEKLDR	17	Intra or Inter
15	82.26	NEVKEESQK	160	VFSSQTTKR	122	Intra or Inter
16	84.48	LKGSNSDSDSPHHASNPHPNSR	65	DFSPVQSQKSQQNR	464	Intra or Inter
17	88.86	QKHQHQTSKSVPR	94	IVDEKLDR	17	Intra or Inter
18	61.21	RQCKCGSANCRGWLFG	478	LDRNGAVKLYR	25	Intra or Inter
19	102.12	HQHQTSKSVPR	94	NGAVKLYR	25	Intra or Inter
20	62.53	VFSSQTTKR	122	LGKTR	154	Intra or Inter
21	82.5	ISKLR	472	LGKTR	154	Intra or Inter
22	60.76	DFSPVQSQKSQQNR	464	HQHQTSKSVPR	94	Intra or Inter
23	69.39	TRNEVKEESQK	160	IVDEKLDR	17	Intra or Inter
24	52.64	EATSPKTSSILTKPR	180	NEVKEESQKR	165	Intra or Inter
25	67.48	EATSPKTSSILTKPR	180	TRNEVKEESQK	160	Intra or Inter
26	81.3	TRNEVKEESQKR	160	TSSILTKPR	187	Intra or Inter
27	57.99	NGAVKLYR	25	LGKTR	154	Intra or Inter
28	86.11	KVFSSQTTK	114	ELNVKK	109	Intra or Inter
29	58.74	KVFSSQTTK	114	ISKLR	472	Intra or Inter
30	52.86	ELNVKK	109	ISKLR	472	Intra or Inter
31	63.42	TRNEVKEESQKR	165	ELVSNSIKEATSPK	174	Intra or Inter
32	70.29	VFSSQTTKR	122	NGAVKLYR	25	Intra or Inter
33	53.32	EKGWGVRSLR	338	TSSILTKPR	187	Intra or Inter
34	62.74	ELVSNSIKEATSPK	174	NEVKEESQKR	160	Intra or Inter
35	78.44	DFSPVQSQKSQQNR	464	VFSSQTTKR	122	Intra or Inter
36	89.46	VFSSQTTKR	122	IVDEKLDR	17	Intra or Inter
37	88.2	KVFSSQTTK	114	NGAVKLYR	25	Intra or Inter
38	53.69	VFSSQTTKR	122	EKGWGVR	338	Intra or Inter
39	57.23	EATSPKTSSILTKPR	180	VFSSQTTKR	122	Intra or Inter
40	54.59	NGAVKLYR	25	ELNVKK	109	Intra or Inter
41	69.86	TSSILTKPR	187	LGKTR	154	Intra or Inter
42	57.32	EESQKRELVSNSIKEATSPK	165	KENKKVFSSQTTK	110	Intra or Inter
43	62.98	KLGKTRNEVKEESQKR	151	QKHQHQTSKSVPR	94	Intra or Inter
44	70.58	RELVSNSIKEATSPK	174	EATSPKTSSILTKPR	180	Inter
45	52.76	NEVKEESQKRELVSNSIKEATSPK	160	KVFSSQTTK	114	Intra or Inter
46	60.42	EESQKRELVSNSIKEATSPK	165	KENKKVFSSQTTKR	122	Intra or Inter
47	67.9	RELVSNSIKEATSPK	174	ELVSNSIKEATSPK	174	Inter
48	61.91	QKHQHQTSKSVPRSQRFSR	87	FSRELNVKKENKK	113	Inter
49	61.91	GGSMSPKQEEYEVERIVDEK	4	KENKKVFSSQTTK	113	Intra or Inter
50	54.39	NPSKLDSYTHLSFYEKRELFR	193	LDSYTHLSFYEKR	205	Inter
51	54.56	GGSMSPKQEEYEVERIVDEKLDR	17	TRNEVKEESQKR	165	Intra or Inter
52	60.27	EATSPKTSSILTKPRNPSK	187	LDSYTHLSFYEKR	205	Intra or Inter
53	53.3	SVPRSQRFSRELNVKKENKK	113	KENKKVFSSQTTKRQSRK	110	Inter
54	60.27	KLGKTRNEVKEESQK	160	LDSYTHLSFYEKR	205	Intra or Inter
55	53.49	EATSPKTSSILTKPR	180	HQHQTSKSVPR	94	Intra or Inter
56	56.23	EESQKRELVSNSIKEATSPK	174	EKGWGVRSLR	338	Intra or Inter
57	57.8	QKHQHQTSKSVPRSQRFSR	87	TKEKGWGVRSLR	336	Intra or Inter
58	58.72	LDSYTHLSFYEKRELFRKK	211	FSRELNVKKENKK	113	Intra or Inter
59	99.46	IVDEKLDR	17	NGAVKLYR	25	Intra or Inter
60	57.18	GGSMSPKQEEYEVER	4	IVDEKLDR	17	Intra or Inter

Supplementary Table S3. List of primers used in this study

Primer Name	Sequence	Description
spClr4_FL_Bam_Fw	GGATCCATGTCGCCTAAACAAGAGGAG	Clr4 expression vector
clr4_62_EcoRI_Rv	GAATTCTTACCTCTTCCGCCTTTTCCATTC	Clr4 expression vector
clr4_126_EcoRI_Rv	GAATTCTTATCGAGATTGTCTCTTGGTAG	Clr4 expression vector
clr4_191_EcoRI_Rv	GAATTCTTAAGGGTTTCGCGGTTTTGTGAG	Clr4 expression vector
clr4_63_BamHI_Fw_2	GGATCCAGACTAAAGGGAAGTAACTCCG	Clr4 expression vector
clr4_127_BamHI_Fw_2	GGATCCAAACAATCCACCGCTCTG	Clr4 expression vector
clr4_83_EcoRI_Rv	GAATTCTTAGTTTGGATGTGGATTGCTCGC	Clr4 expression vector
clr4_104_EcoRI_Rv	GAATTCTTACCGCGAAAATCTTTGAGAACG	Clr4 expression vector
pMALc2_TEV-clr4 192-E Fw	ATCTTTATTTTCAAGGTGGATCCTCCAAACTTGACTC	Clr4 expression vector
pMALc2_TEV-clr4 192-E Rv	CGACGGCCAGTGCCAAGCTTGCCTGCAGGTCGAGCG	Clr4 expression vector
pMAL seq	AACCGCTGGGTGCCGTAGCGCTGAA	Clr4 expression vector
T7	TAATACGACTCACTATAGGG	Clr4 expression vector
SP6	ATTTAGGTGACACTATAG	Clr4 expression vector
pGEX-6P_Seq_Rev	CGTCTCCGGGAGCTGCATGTGTCAG	Clr4 expression vector
TEV_BamHI_clr4_GA_Fw1	TATACTTCCAAGGCCTCGAGGGATCCATGTCGCCT	Clr4 expression vector
TEV_BamHI_clr4_192-END_GA_Fw1	TATACTTCCAAGGCCTCGAGGGATCCTCCAAACTTGAC	Clr4 expression vector
clr4END_HindIII_GA_Rv1	TATCTAGACTGCAGGTCGACAAGCTTGCCTGCAGG	Clr4 expression vector
Clr4_C_Fw	TTTAGACATGTTTGATGATGCTAGC	Clr4 mutagenesis
Clr4_C_Rv	CGGCCAGTGCCAAGCTTGCCTGCAG	Clr4 mutagenesis
Clr4_K455A	GATTACGCTGGTGCAGCAGATTTCTCACCTG	Clr4 mutagenesis
Clr4_K472A	GCAAAATAGAATTTCAGCACTTCGCCGGCAG	Clr4 mutagenesis
Clr4_K455A_Rv	GCTGCACCAGCGTAATCAAATG	Clr4 mutagenesis
Clr4_K472A_Rv	GCTGAAATTCTATTTTGCTGAGA	Clr4 mutagenesis
Clr4_K464A/R469A_Rv1	GCATTTTGCTGAGATGCTTGAGATTGAACAGGTGAG	Clr4 mutagenesis
Clr4_K464A/R469A_Fw1	CAAGCATCTCAGCAAAATGCAATTTCAGCACTTCGC	Clr4 mutagenesis
Clr4_C_QA_Rv1	ATTCTATTTTGCTGCAGATTTTGCAGATGCAACAGGTGAG	Clr4 mutagenesis
Clr4_C_QA_Fw2	TGCATCTGCAAAATCTGCAGCAAATAGAATTTCAAAACTTCGCCG	Clr4 mutagenesis
Clr4_d458-467_Rv	AAATTCTATTGAAATCTTTTGCACCAGCG	Clr4 mutagenesis
Clr4_d458-467_Fw	AAAAGATTTCAATAGAATTTCAAAACTTCGCCGGCAG	Clr4 mutagenesis
Clr4_ARL_DIM-5_Fw2	AATGGTCTTACGGGTCTTGAGTCTGATGCTCATGATCCTTCTAAAATTTCTGAGCTTCGCCGGCAGTGCAAAT	Clr4 mutagenesis
Clr4_ARL_DIM-5_Rv1	CTCAGAAATTTTAGAAGGATCATGAGCATCAGACTCAAGACCCGTAAGACCATTAGCGTAATCAAATGTAAGCTCC	Clr4 mutagenesis
Clr4_ARL_GS_Fw2	GGTGGTGGTGGTTCTGGAGGAGGAGGATCCGGTGGAGGTGGATCCGGAGGTGGTGGATCTCTTCGCCGGCAGTGCAAAT	Clr4 mutagenesis
Clr4_ARL_GS_Rv1	AGATCCACCACCTCCGGATCCACCTCCACCGGATCCTCCTCCTCCAGAACCACCACCACCAGCGTAATCAAATGTAAGC	Clr4 mutagenesis
Clr4_472-475A_Fw_GA	GCCGCCGCCGCCCAGTGCAAATGTGGTTC	Clr4 mutagenesis
Clr4_472-475A_Rv_GA	TGCACTGGGCGGCGGCGGCTGAAATTCTATTTTGCTGAG	Clr4 mutagenesis
Clr4_472-475E_Fw_GA	GAGGAGGAGGAGCAGTGCAAATGTGGTTC	Clr4 mutagenesis
Clr4_472-475E_Rv_GA	TGCACTGCTCCTCCTCCTCTGAAATTCTATTTTGCTGAG	Clr4 mutagenesis
Clr4N1_GA_UP_Fw2	TCTGTTCCAGGGGCCCCTGGGATCC	Clr4 mutagenesis
clr4_XhoI	GTCACGATGCGGCCGCTCGAG	Clr4 mutagenesis
clr4N_S_Rv1_GA	GACGAAGAGGAGTTACTTCCAGATAGTGACCTCTTCCGCCTTTTCCATTC	Clr4 mutagenesis
clr4N_S_Fw1_GA	TCACTATCTGGAAGTAACTCCTCTTCGTCTTCACCGCACCATGCGAGC	Clr4 mutagenesis
clr4N_S_Rv2_GA	TCATCAGCTACATCCCCCTCAACATCTTCAATTCTCACATCACCGTCAAACCCTTAAGAATTCCCGG	Clr4 mutagenesis
clr4N_S_Fw2_GA	GACGGTGATGTGAGAATTGAAGATGTTGAGGGGGATGTAGCTGATGAAATAGAATTGGAAACTAG	Clr4 mutagenesis
clr4N_S_Fw4_GA	AGTACTAGTATCATTTTATCATCTAGTCTTCATACAAATTCATCTTCACTGGGGAAGACGAGAAATG	Clr4 mutagenesis
clr4N_S_Rv4_GA	TGAAGATGAATTTGTATGAAGACTAGATGATAAAATGATACTAGTACTATTCGTCGTCAGAGCGG	Clr4 mutagenesis
clr4N_S_Fw5_GA	TCATCGTCAAATTCATCTGTTTTTTCTTCTCAAACTACCTCTTCACAATCTTCTTCACAATCCACCGCTCTGA	Clr4 mutagenesis
clr4N_S_Rv5_GA	TGAAGAAGATTGTGAAGAGGTAGTTTGAGAAGAAAAAACAGATGAATTTGACGATGATACATTCAATTCCCGCG	Clr4 mutagenesis
clr4N_S_Fw3_GA2	TCACAATCTCACCAACATCAAACTTCATCTTCAGTGCCTTCTTCTCAATCATTTTCGTCTTCATTGAATGTAAAAAAGGA	Clr4 mutagenesis
clr4N_S_Fw3_GA	TCACAATCTCACCAACATCAAACTTCATCTTCAGTGCCTTCTTCTCAATCATTTTCGTCTTCATTGAATGTATCATCGTC	Clr4 mutagenesis
clr4N_S_Rv3_GA	TGAAGACGAAAATGATTGAGAAGAAGGCACTGAAGATGAAGTTTGATGTTGGTGAGATTGTGAACTGTTTGGATGTGGA	Clr4 mutagenesis
clr4N_S_Fw6_GA2	TCTACGTCAAATTCAGTGTCTTCTTCATCTCAATCATCATCTCTAGTTTCCAATTCTAT	Clr4 mutagenesis
clr4N_S_Rv6_GA2	AGATGATGATTGAGATGAAGAAGACACTGAATTTGACGTAGACCCCAGCTTCTTTGA	Clr4 mutagenesis
Clr4IDR_BQ_GA_UP_Rv	TGCGAAAACTGTTGAGACTGAGGCACTGACTGTGAAGTTTGATGTTGGTGCTGTTGCTGACTGTTTGGATGTGGA	Clr4 mutagenesis
Clr4IDR_BQ_GA_DW_Fw	CAGCAACAGCACCAACATCAAACTTCACAGTCAGTGCCTCAGTCTCAACAGTTTTCGCAGGAATTGAATGTACAGC	Clr4 mutagenesis
Clr4IDR_CQ_GA_UP_Rv	CTGCTGAGATTGCTGCTGGGTAGTTTGAGAAGAAAAAACCTGCTGATTTTCCTGCTGTACATTCAATTCCCGCG	Clr4 mutagenesis
Clr4IDR_CQ_GA_DW_Fw	CAGCAGGAAAATCAGCAGGTTTTTTCTTCTCAAACTACCCAGCAGCAATCTCAGCAGCAATCCACCGCTCTGA	Clr4 mutagenesis
Clr4IDR_BQ_GA_DW_Fw2	CAGCAACAGCACCAACATCAAACTTCACAGTCAGTGCCTCAGTCTCAACAGTTTTCGCAGGAATTGAATGTAAAAAAGGA	Clr4 mutagenesis
clr4_NheI_GA_Rv	ATCCACTGTATACTCGCTAGC	Clr4 strain construction
TOPO_BamHI_GA_Fw	GCTTGGTACCGAGCTCGGATCC	Clr4 strain construction
clr4_BamHI_GA_Fw	CTTTATTTTCAAGGTGGATCC	Clr4 strain construction
Clr4_Pr_GA_Fw	CCAGCTGAAGCTTCGTACGCTGCAGGTCTTCATTAGCCAGCGTCTATGT	Clr4 strain construction
Clr4_Pr_Fw2	GTCTTCATTAGCCAGCGTCTATGT	Clr4 strain construction
Clr4_END_GA_Rv	ATAAGAAATTCGCTTATTTAGAAGTTTAACCGAAAAGCCAGCCACGACA	Clr4 strain construction
Clr4_DW_GA_Fw	AACGCCGCCATCCAGTTTAAACGAGGCTAATTTATTACTGGTTGACTCCA	Clr4 strain construction
Clr4_DW_GA_Rv	CGCATAGGCCACTAGTGGATCTGATGCAGATCCTTCCCTTGAATTTGCT	Clr4 strain construction
Clr4_DW_RV2	GCAGATCCTTCCCTTGAATTTGCT	Clr4 strain construction
meu6_UP_GA_Fw	GAAGCTTCGTACGCTGCAGGTCGACGGAACTAATGGTGACAATCATGT	Clr4 strain construction
meu6_UP_Fw2	GGAACTAATGGTGACAATCATGT	Clr4 strain construction
meu6_UP_GA_Rv	GGGACGAGGCAAGCTAAACAGATCTCGAAAACAACAGTTAGGCTCTCA	Clr4 strain construction
meu6_DW_GA_Fw	CCGCCATCCAGTTTAAACGAGCTCGAAGAAGCTCTCAGGTTTGGTGCA	Clr4 strain construction
meu6_DW_GA_Rv	CGCATAGGCCACTAGTGGATCTGATGCTAATTTATTACTGGTTGACTCC	Clr4 strain construction
meu6_DW_Rv2	GCTAATTTATTACTGGTTGACTCC	Clr4 strain construction
Clr4_Pr_Fw3	GTCTTCATTAGCCAGCGTCTATGTA	Clr4 strain construction
meu6_UP_Fw3	GGAACTAATGGTGACAATCATGTA	Clr4 strain construction
clr4_dw_GA_Rv	CTAACGCCGCCATCCAGTTTAAAC	Clr4 strain construction
clr4_pREP_GA_Fw	CCCATATGTCGACTCTAGAGGGATCCATGTCGCCTAAAC	Clr4 nuclear localization check
clr4_pREP_GA_Rv	TACCTATTTACCCGGGGATCTTAACCGAAAAGCCAGC	Clr4 nuclear localization check
spClr4-UP-check	ATTACTTTGTACTTGATACC	*clr4* deletion
spClr4-DW-Rv	GCTCCTTCTATAGCTTCTGAAGG	*clr4* deletion

rik1/END/FW	AGTCGACACGGAAATTGGTGCTATTGGGAG	*rik1* tagging
rik1/DW/RV	CTGATATCCTCTGACCCTAGCTTCCT	*rik1* tagging
ago1/UP/FW	CGTTTGCGTGCTCAGAGAAGT	*ago1* deletion
ago1/dw-RV	CGATATCGCATTCCGTATAGGTCGCTCATC	*ago1* deletion
spAtf1-UP-Fw	CTGTACAGACATCTAACTAATTC	*atf1* deletion
atf1-UP-Rv-x	GGGGATCCGTCGACCTGCAGCGTACGATGAAGAATTTATGCTTTAACACTTGAC	*atf1* deletion
spAtf1-DW-Fw-y	AAACGAGCTCGAATTCATCGATGAATGCATTTAGCTATGTTATGG	*atf1* deletion
spAtf1-DW-Rv	CTCCCACAATACACTAAGAC	*atf1* deletion
SpPcr1-FW(-455)	TTGAAGTCGTTTGGGATCTCC	*pcr1* deletion
pcr1-UP-Rv-x	GGGGATCCGTCGACCTGCAGCGTACGACTTAAATAATCAATGTATAG	*pcr1* deletion
pcr1-DW-Fw-y	AAACGAGCTCGAATTCATCGATGACTCTCCTAGATGACAGTCGG	*pcr1* deletion
pcr1-DW-Rv	CACAGTTGCTCCAACCAGAG	*pcr1* deletion
act1 RT-Fw1	CGTGCCCCTGAAGCTCTTT	qPCR
act1 RT-Rv1	CTCATGAATACCGGCGTTTTC	qPCR
SpCen-dgF-Fw	CTGCGGTTCACCCTTAACAT	qPCR
SpCen-dgF-Rv	CAACTGCGGATGGAAAAAGT	qPCR
cenH dh Rv	GCTAAGATCGATTGGTGACG	qPCR
cenH dh Fw	AAGTTCACTGTTCTTATACACTGG	qPCR
ade6-qPCR-Fw	GGATGCAGCAAATTCTCCTGCC	qPCR
ade6-qPCR-Rv	GACAGTTCAACAATTGCATCGGGG	qPCR
ade6-DN/N Fw	GTAGTACGCAGTTTAGACGG	qPCR
ade6-DN/N Rv	GAGCACGCTGTTGAATTGAG	qPCR
ade6-qPCR-Rv2	GTAGCAAGTATACGACAGGC	qPCR
ade6_P489_Fw2	CTCTATTGTTCAGATGCC	qPCR
ura4 RT-Fw1	GGCCTCAAAGAAGTTGGTTTACC	qPCR
ura4 RT-Rv1	GAAGACATTTCAGCCAAAAGCA	qPCR
Sp_SIRE1/4_Fw(+GGG)	GGGAGCCTCAAGTGACTGCATTAAAG	EMSA (DNA)
T7-Sp_SIRE1/4_Fw	TAATACGACTCACTATAGGGAGCCTCAAGTGACTGCATTAAAG	*in vitro* transcription
Sp_SIRE4/4_Rv	GGGCTACTCTGAAGACAACGA	*in vitro* transcription
T7-SpUra4ssRNA2/2_F	TAATACGACTCACTATAGGGTACCTTTGGGACGTGGTCTC	*in vitro* transcription
Sp_Ura4ssRNA1/2_Rv	CGACCAGCTCCATAGACTCC	*in vitro* transcription

Supplementary Table S4. List of S. pombe strains used in this study

Strain name	Genotype
PG3464	h^{90} ade6-DN/N ura4-D18 leu1-32 mat3-M::ade6^{+}
SPRN118	h^{90} ade6-DN/N ura4-D18 leu1-32 mat3-M::ade6^{+} clr4Δ::nat^{R}
SPRN58	h^{90} ade6-DN/N ura4-D18 leu1-32 mat3-M::ade6^{+} clr4::5xFlag-clr4^{WT} meu6Δ::kan^{R}
SPRN91	h^{90} ade6-DN/N ura4-D18 leu1-32 mat3-M::ade6^{+} clr4::5xFlag-clr4^{mut1} meu6Δ::kan^{R}
SPRN93	h^{90} ade6-DN/N ura4-D18 leu1-32 mat3-M::ade6^{+} clr4::5xFlag-clr4^{mut2} meu6Δ::kan^{R}
SPRN81	h^{90} ade6-DN/N ura4-D18 leu1-32 mat3-M::ade6^{+} clr4::5xFlag-clr4^{mut3} meu6Δ::kan^{R}
SPRN105	h^{90} ade6-DN/N ura4-D18 leu1-32 mat3-M::ade6^{+} ago1Δ::hyg^{R}
SPRN128	h^{90} ade6-DN/N ura4-D18 leu1-32 mat3-M::ade6^{+} clr4Δ::nat^{R} ago1Δ::hyg^{R}
SPRN107	h^{90} ade6-DN/N ura4-D18 leu1-32 mat3-M::ade6^{+} clr4::5xFlag-clr4^{WT} meu6Δ::kan^{R} ago1Δ::hyg^{R}
SPRN109	h^{90} ade6-DN/N ura4-D18 leu1-32 mat3-M::ade6^{+} clr4::5xFlag-clr4^{mut1} meu6Δ::kan^{R} ago1Δ::hyg^{R}
SPRN111	h^{90} ade6-DN/N ura4-D18 leu1-32 mat3-M::ade6^{+} clr4::5xFlag-clr4^{mut2} meu6Δ::kan^{R} ago1Δ::hyg^{R}
SPRN112	h^{90} ade6-DN/N ura4-D18 leu1-32 mat3-M::ade6^{+} clr4::5xFlag-clr4^{mut3} meu6Δ::kan^{R} ago1Δ::hyg^{R}
SPM1606	h^{+} ade6-210 ura4-D18 leu1-32 otr1R::ade6^{+}
SPRN103	h^{+} ade6-210 ura4-D18 leu1-32 otr1R::ade6^{+} rik1::rik1-13myc-hyg^{R}
SPRN75	h^{+} ade6-210 ura4-D18 leu1-32 otr1R::ade6^{+} clr4::5xFlag-clr4^{WT} meu6Δ::kanR rik1::rik1-13myc-hyg^{R}
SPRN99	h^{+} ade6-210 ura4-D18 leu1-32 otr1R::ade6^{+} clr4::5xFlag-clr4^{mut1} meu6Δ::kan^{R} rik1::rik1-13myc-hyg^{R}
SPRN101	h^{+} ade6-210 ura4-D18 leu1-32 otr1R::ade6^{+} clr4::5xFlag-clr4^{mut2} meu6Δ::kan^{R} rik1::rik1-13myc-hyg^{R}
SPRN79	h^{+} ade6-210 ura4-D18 leu1-32 otr1R::ade6^{+} clr4::5xFlag-clr4^{mut3} meu6Δ::kan^{R} rik1::rik1-13myc-hyg^{R}
SPYB106	h^{90} ade6-216 ura4-DS/E leu1-32 his2 Kint2::ura4^{+}
SPM80	h^{90} ade6-216 ura4-DS/E leu1-32 his2 Kint2::ura4^{+} clr4Δ::kan^{R}
SPRN121	h^{90} ade6-216 ura4-DS/E leu1-32 his2 Kint2::ura4^{+} clr4::5xFlag-clr4^{WT} meu6Δ::kan^{R}
SPRN123	h^{90} ade6-216 ura4-DS/E leu1-32 his2 Kint2::ura4^{+} clr4::5xFlag-clr4^{mut1} meu6Δ::kan^{R}
SPRN126	h^{90} ade6-216 ura4-DS/E leu1-32 his2 Kint2::ura4^{+} clr4::5xFlag-clr4^{mut2} meu6Δ::kan^{R}
SPRN127	h^{90} ade6-216 ura4-DS/E leu1-32 his2 Kint2::ura4^{+} clr4::5xFlag-clr4^{mut3} meu6Δ::kan^{R}
SPM1626	h^{+} ade6-210 ura4-D18 leu1-32 otr1R::ade6^{+} clr4Δ::kan^{R}
SPRN73	h^{+} ade6-210 ura4-D18 leu1-32 otr1R::ade6^{+} clr4::5xFlag-clr4^{WT} meu6Δ::kan^{R}
SPRN95	h^{+} ade6-210 ura4-D18 leu1-32 otr1R::ade6^{+} clr4::5xFlag-clr4^{mut1} meu6Δ::kan^{R}
SPRN97	h^{+} ade6-210 ura4-D18 leu1-32 otr1R::ade6^{+} clr4::5xFlag-clr4^{mut2} meu6Δ::kan^{R}
SPRN77	h^{+} ade6-210 ura4-D18 leu1-32 otr1R::ade6^{+} clr4::5xFlag-clr4^{mut3} meu6Δ::kan^{R}
SPRN132	h^{+} ade6-210 ura4-D18 leu1-32 otr1R::ade6^{+} clr4Δ::kan^{R} ago1Δ::hyg^{R}
SPRN139	h^{+} ade6-210 ura4-D18 leu1-32 otr1R::ade6^{+} clr4::5xFlag-clr4^{WT} meu6Δ::kan^{R} ago1Δ::hyg^{R}
SPRN134	h^{+} ade6-210 ura4-D18 leu1-32 otr1R::ade6^{+} clr4::5xFlag-clr4^{mut1} meu6Δ::kan^{R} ago1Δ::hyg^{R}
SPRN136	h^{+} ade6-210 ura4-D18 leu1-32 otr1R::ade6^{+} clr4::5xFlag-clr4^{mut2} meu6Δ::kan^{R} ago1Δ::hyg^{R}
SPRN137	h^{+} ade6-210 ura4-D18 leu1-32 otr1R::ade6^{+} clr4::5xFlag-clr4^{mut3} meu6Δ::kan^{R} ago1Δ::hyg^{R}
SPRN180	h^{90} ade6-DN/N ura4-D18 leu1-32 mat3-M::ade6^{+} clr4Δ::nat^{R} atf1Δ::hyg^{R}
SPRN178	h^{90} ade6-DN/N ura4-D18 leu1-32 mat3-M::ade6^{+} clr4::5xFlag-clr4^{WT} meu6Δ::kan^{R} atf1Δ::hyg^{R}
SPRN182	h^{90} ade6-DN/N ura4-D18 leu1-32 mat3-M::ade6^{+} clr4::5xFlag-clr4^{mut1} meu6Δ::kan^{R} atf1Δ::hyg^{R}
SPRN184	h^{90} ade6-DN/N ura4-D18 leu1-32 mat3-M::ade6^{+} clr4::5xFlag-clr4^{mut2} meu6Δ::kan^{R} atf1Δ::hyg^{R}
SPRN186	h^{90} ade6-DN/N ura4-D18 leu1-32 mat3-M::ade6^{+} clr4::5xFlag-clr4^{mut3} meu6Δ::kan^{R} atf1Δ::hyg^{R}
SPRN170	h^{90} ade6-DN/N ura4-D18 leu1-32 mat3-M::ade6^{+} clr4Δ::nat^{R} pcr1Δ::hyg^{R}
SPRN168	h^{90} ade6-DN/N ura4-D18 leu1-32 mat3-M::ade6^{+} clr4::5xFlag-clr4^{WT} meu6Δ::kan^{R} pcr1Δ::hyg^{R}
SPRN172	h^{90} ade6-DN/N ura4-D18 leu1-32 mat3-M::ade6^{+} clr4::5xFlag-clr4^{mut1} meu6Δ::kan^{R} pcr1Δ::hyg^{R}
SPRN174	h^{90} ade6-DN/N ura4-D18 leu1-32 mat3-M::ade6^{+} clr4::5xFlag-clr4^{mut2} meu6Δ::kan^{R} pcr1Δ::hyg^{R}
SPRN176	h^{90} ade6-DN/N ura4-D18 leu1-32 mat3-M::ade6^{+} clr4::5xFlag-clr4^{mut3} meu6Δ::kan^{R} pcr1Δ::hyg^{R}
